# Correlation-maximizing surrogate gene space for visual mining of gene expression patterns in developing barley endosperm tissue

**DOI:** 10.1186/1471-2105-8-165

**Published:** 2007-05-22

**Authors:** Marc Strickert, Nese Sreenivasulu, Björn Usadel, Udo Seiffert

**Affiliations:** 1 Leibniz Institute of Plant Genetics and Crop Plant Research (IPK), D-06466 Gatersleben, Germany; 2 Max-Planck-lnstitute of Molecular Plant Physiology, 14424 Potsdam, Germany

## Abstract

**Background:**

Micro- and macroarray technologies help acquire thousands of gene expression patterns covering important biological processes during plant ontogeny. Particularly, faithful visualization methods are beneficial for revealing interesting gene expression patterns and functional relationships of coexpressed genes. Such screening helps to gain deeper insights into regulatory behavior and cellular responses, as will be discussed for expression data of developing barley endosperm tissue. For that purpose, high-throughput multidimensional scaling (HiT-MDS), a recent method for similarity-preserving data embedding, is substantially refined and used for (a) assessing the quality and reliability of centroid gene expression patterns, and for (b) derivation of functional relationships of coexpressed genes of endosperm tissue during barley grain development (0–26 days after flowering).

**Results:**

Temporal expression profiles of 4824 genes at 14 time points are faithfully embedded into two-dimensional displays. Thereby, similar shapes of coexpressed genes get closely grouped by a correlation-based similarity measure. As a main result, by using power transformation of correlation terms, a characteristic cloud of points with bipolar sandglass shape is obtained that is inherently connected to expression patterns of pre-storage, intermediate and storage phase of endosperm development.

**Conclusion:**

The new HiT-MDS-2 method helps to create global views of expression patterns and to validate centroids obtained from clustering programs. Furthermore, functional gene annotation for developing endosperm barley tissue is successfully mapped to the visualization, making easy localization of major centroids of enriched functional categories possible.

## Background

The essence of gene expression analysis is similarity-based screening and structuring of hybridization data. Several methods exist to realize the workflow of raw array data preprocessing, background correction, filtering, clustering and/or classification to identify preferentially expressed genes and to recognize over-represented functional groups using annotation information [1,2]. The quality of each step in that processing pipeline should be validated, though. In this work, a faithful visualization technique for comparative data displays is presented for assisting in validation. Typical questions arising during expression analysis, addressed by such visualization, are: on one hand, how are hybridization experiments related to each other, and are replication experiments consistent with previously taken data? On the other hand, can correspondence be found between gene-specific expression patterns, and are centroids of gene expressions – such as obtained from k-means or neural gas clustering – located appropriately? Last not least, can typical data clusters be identified by appropriate display, either for experiments or for gene expression patterns?

Principal component analysis (PCA) – often realized as singular value decomposition (SVD) – is the standard technique to create low-dimensional displays of high-dimensional data [3]. Once eigenvectors are calculated, fast linear mappings on the principal components are possible that explain directions of maximum variance. Thereby, Euclidean data space is implicitly assumed for variance maximization. The restriction of PCA to linear mappings of Euclidean spaces can be overcome by using more general multidimensional scaling (MDS) approaches. These assign each high-dimensional data point a low-dimensional counterpart and minimize the discrepancy of the points' relationships in high- and low-dimensional space. High-dimensional input data, for example, might be compared by Minkowski metrics or by Pearson correlation similarity. The low-dimensional output space should be Euclidean – this allows a visual interpretation of close points as representing similar input data, and distant points as indicating dissimilarities. Since, for such view, *high *similarity is expressed by *small *values and vice versa, this inverse interpretation is sometimes referred to as dissimilarity in the literature.

For gene expression data, *correlation *similarity is very useful, because dense clusters of displayed points then do coincide with highly correlated expression vectors. In coexpression-related analysis, time series of gene expressions should be clustered if their temporal profiles are similar, while discorrelated dynamics should be separated. Hierarchical clustering [4], k-means [5], and self-organizing maps (SOM) [6] usually facilitate the grouping task. Some problems remain, though: in hierarchical clustering the resulting ordering is not unique and the corresponding large tree is difficult to access visually; both k-means and SOM induce data abstractions by setting a debatable number of centroids; by choosing additional free parameters for the architecture and learning process, SOM can be used for cluster visualization, but faithful SOM training requires an appropriate choice of parameters – only then, similar clusters do commonly correspond to adjacent SOM centroids. Since the vector quantization in SOM provides a mapping of input vectors to a corresponding centroid, their individuality gets lost which complicates outlier identification. Other authors have pointed out the need for a visual inspection of the gene space for comparison and validation of clustering results. The microarray latent visualization and analysis package (MILVA) is designed for mapping the gene space to a two-dimensional display using either generative topographic mapping (GTM) or the NeuroScale method [7]. Due to its built-in functional mapping, the software is very well suited for smooth interactive gene explorations. However, it requires prior assumptions to estimate density models from the available high-dimensional data for characterizing the underlying data manifold. An embedding technique for dealing with non-metric data relationships is nMDS [8]. This fast multidimensional scaling approach relies on heuristic reconstruction of rank relationships between input data and their corresponding points in the two-dimensional display. These existing data visualization tools are very useful for interacting with the data. Still, there is further need to improve data displays, especially in gene expression studies, for extracting reliable sets of coexpressed genes and for visually assessing relationships between functional categories of coexpressed genes.

A first version of high-throughput multidimensional scaling (HiT-MDS), realizing metric MDS based on a mathematical cost function formulation, has been proposed in the authors' previous work for Euclidean gene space reconstruction [9]. In a more recent study [10], a comparison of HiT-MDS to an algebraic MDS approach and to the free XGvis system [11] is given. It turns out that it is generally problematic to compare a method optimized for a specific cost minimization with a method aiming at other visualization cost criteria. Thus, a pragmatic rating is 'value by usefulness' which strongly depends on biologically informative displays and somewhat also on computing time. In the present study, two substantial extensions of HiT-MDS are described leading to HiT-MDS-2: one extension corresponds to an improvement of the MDS cost function without changing the original embedding quality, the other corresponds to the utilization of non-Euclidean measures for input data, namely, powers of Pearson correlation, for the visual exploration of regulatory patterns in temporal gene expression profiles. Here, we demonstrate the HiT-MDS-2 tool for improved assessment of quality and reliability of centroids of temporal gene expression profiles, and for pointing out visual relationships between functional categories of coexpressed genes. This allows to identify robustly the key regulatory genes in sets of transcriptionally co-regulated genes, such as from developing endosperm tissue in barley.

## Results

### Data of developing barley endosperm tissue

In order to demonstrate its benefits, the presented HiT-MDS-2 algorithm has been applied to an expression data set obtained from a 12 k seed array (11786 genes) of developing barley grains [12]. The pursued hybridization experiments produced comprehensive transcriptome data covering all major events of endosperm development from 14 time points corresponding to a time span of 0 to 26 days after flowering (DAF), in two day intervals. The HiT-MDS-2 algorithm is used to address three major questions: 1. How are the experiments, representing transient development of endosperm tissue, characterized with respect to their transcriptome similarity of specifically expressed genes? 2. Which are the main regulatory genes, represented in a set of transcriptionally co-regulated genes in developing endosperm? And, finally, 3. what is their role in explaining temporal differentiation of endosperm tissue?

The 12 k gene expression data set, prepared as discussed in the methods section, is considered from its two fundamental views, one corresponding to individual hybridization experiments each involving 4824 filtered genes, the other corresponding to individual genes with expression values sampled at 14 time points. The embedding-based analysis is thus carried out for (a) experiment grouping and (b) gene profile inspection. Supplemental material is online available [13].

### Experiment grouping

**Figure 1:**
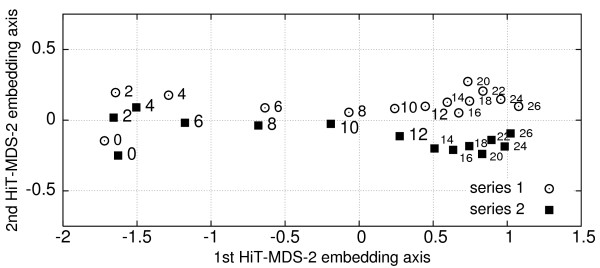
**Embedded relationships of cDNA array experiments**. HiT-MDS-2 visualization of inter-relationships between cDNA array experiments from two independent series of developing barley endosperm tissue. Experiments with 4824 selected log_2_-normalized genes are compared by (1-Pearson correlation)^*p *^at power *p *= 1. Numbers denote days after flowering (DAF). From left to right a clear temporal order is found, corresponding to pre-storage (0–4 DAF), intermediate (6–12 DAF), and storage (14–26 DAF) phases of endosperm development. Day zero, related to the fertilization event, is systematically separated from rest of the early stages. While a relative delay of roughly two days is found between both experimental series during intermediate stages, late stages become more tightly linked (14–26 DAF). Embedding axis 2 separates the two series. Slight systematic differences of series 1 and 2 result from low and high phosphor image scanning resolutions, respectively, and thus from different dynamic ranges.

Visual experiment validation is obtained by embedding their pairwise correlations (1 - **r**(**x**^*i*^, **x**^*j*^)), where **x**^*i *^and **x**^*j *^are experiments *i *and *j*, each containing expression values of 4824 genes. The scatter plot given in Fig. 1 was calculated within 0.5s on a 3 GHz P4 processor with 750 cycles of the data set. The inter-distances of the displayed points correlate at a very high level of rL2(D^,D)=0.990
 MathType@MTEF@5@5@+=feaafiart1ev1aaatCvAUfKttLearuWrP9MDH5MBPbIqV92AaeXatLxBI9gBaebbnrfifHhDYfgasaacH8akY=wiFfYdH8Gipec8Eeeu0xXdbba9frFj0=OqFfea0dXdd9vqai=hGuQ8kuc9pgc9s8qqaq=dirpe0xb9q8qiLsFr0=vr0=vr0dc8meaabaqaciaacaGaaeqabaqabeGadaaakeaaieqacqWFYbGCdaqhaaWcbaacbaGae4htaWeabaGaeGOmaidaaOGaeiikaGIaf8hraqKbaKaacqGGSaalcqWFebarcqGGPaqkcqGH9aqpcqaIWaamcqGGUaGlcqaI5aqocqaI5aqocqaIWaamaaa@3AEF@ with the inter-similarities of the original input data. Thus, the visualization represents almost perfectly the relationships in the 4824-dimensional correlation space of the input data. After display normalization, the zero origin demarcates a critical point for the interpretation of symmetry breaks. As a result, axis 1 can be easily associated with temporal development, axis 2 corresponds to systematic differences in both independent series (Fig. 1). The time domain can be described as follows: (i) the initial experiments at 0 DAF are not in the same line as subsequent time points – this slight orthogonal displacement corresponds to the early fertilization event with its unique gene expression, (ii) transcriptional changes during pre-storage phase are slow until day 4, (iii) between 6 to12 DAF (intermediate phase) a strong transcriptional reprogramming takes place, and (iv) the late stage of 16 to 26 DAF (storage phase) is characterized by a saturation process, indicated by a higher point density on the right, with diminishing transcriptional regulation.

Although embedded experiments are arranged in a consistent manner, not showing major outliers, series 2 is further considered in the following: it exhibits a smoother temporal transition between 6 DAF to 12 DAF than series 1, and, in addition, a better dynamic signal range was found, because the underlying phosphor images were scanned at higher resolution than those of series 1.

### Gene profile inspection

#### HiT-MDS-2 scatter plots for the visual validation of clusters of gene expression patterns

**Figure 2:**
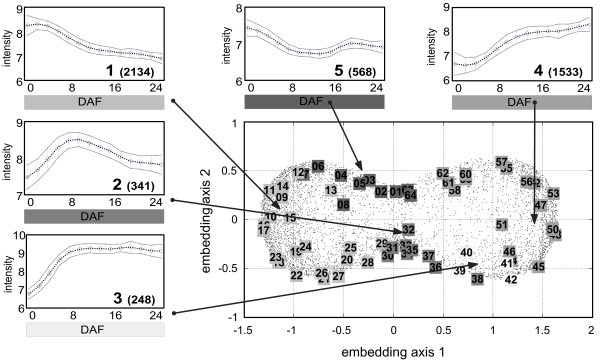
**Gene expression correlation space obtained by HiT-MDS-2**. Surrogate gene space of developing barley endosperm tissue. A total number of 4824 temporally regulated, log-normalized gene profiles with 14 time points are embedded using powers of *p *= 8 of Pearson correlation for profile comparison. The bipolar sandglass shape of points is labeled by 64 centroids obtained from correlation-based neural gas (NG) clustering which yields a good density-related covering of the data space. The clusters fall into five biologically meaningful regulatory different patterns: each of the five small panels displays the average and standard deviation of aligned genes belonging to the marked centroids – the number of genes within each category 1–5 are given in parentheses. The HiT-MDS-2 embedding shows a consistent spatial arrangement of clusters within the manually selected categories (apart from clusters 8, 13, and 51); pre-storage phase (cluster group 1), intermediate phase (cluster groups 2 and 5, anti-correlated to each other), and storage phase (cluster groups 3 and 4). Since the exponent *p *= 8 magnifies small differences, the resulting large spatial variability for down- and up-regulation represented by cluster groups 3 and 5 do still correspond to only small standard deviations. The small asymmetric bias of the gene cloud to the right indicates more subtle patterns found in up-regulation rather than in down-regulation, which underlines the benefit of the visualization for screening and validation.

Dealing with thousands of temporally regulated genes is a crucial task. Tools for intuitive inspection of the gene space help to identify coexpressed gene sets associated with biological processes occurring during development. The ESTs selected for the 12 k seed array fabrication were taken from cDNA libraries specific to pre-storage and storage phase of developing seeds. This selection leads to pronounced temporal gene regulation, which results in a bipolar sandglass shape in the corresponding HiT-MDS-2 display of embedded expression data. This shape represents genes with up- and down-regulation, corresponding to pre-storage and storage phase, respectively (Fig. 2). Start and end of development, and the temporal transition between the phases have been characterized in Fig. 1 in the previous section. As explained below, by using Fig. 2 a set of 340 genes with intermediate regulation can be detected, which is responsible for the observed transition event.

The sandglass shape with its well-spread points results from power transformations of correlations, which magnifies subtle dissimilarities in highly correlated genes. In the presence of many coexpressed genes, powers applied to the input similarities shift the corresponding histogram towards zero; this leads to focus on a good reconstruction – and thus a fair differentiation – of highly correlated, i.e. with near zero dissimilarities, rather than of obviously discorrelated genes. A power of *p *= 8 applied to the input similarities, i.e. (1 - **r**(**x**^*i*^, **x**^*j*^))^8^, is a good choice for clearly separating between up- and down regulated expression patterns during pre-storage and storage phase. Setting *p *= 8 is a compromise for spreading highly correlated genes and for giving space also to intermediate regulations. Comparative density plots for exponents of *p *= 24, 4,1, 0.25 are available extra [see Additional file 1]. These results indicate how powers of correlations help to emphasize the specific relationship structure in the set of genes. Similar findings are also reported by Zhou et al. for shortest path analysis in gene expression data [14].

For the number of 4824 genes, 100 data cycles are sufficient to get a high-quality display shown in Fig. 2. Overall, the HiT-MDS-2 embedding procedure applied to transcriptome data of endosperm development yields a faithful arrangement of genes with their typical temporal expressions. These are clearly divided into sets with expressions of pre-storage (cluster group 1), intermediate (cluster group 2), and storage phase (cluster group 3 and 4). The corresponding temporal expression patterns are revealed by browsing the scatter plot from the left to the right side of the bipolar sandglass shape (Fig. 2). In addition, we also noticed very interesting patterns showing dominant expression values in the pre-storage phase with drastic decrease in the intermediate stage, followed by an increase of expression levels during the storage phase (Fig. 2, cluster group 5). These results indicate that the non-linear data embedding technique of HiT-MDS-2 is a useful tool for identifying not only the major global patterns occurring during temporal development; also informative minor patterns that could be easily missed in noisy subsets of gene expression data show up as scattered point sets.

We further examined whether the non-linear 2D representation of the gene space obtained by HiT-MDS-2 is also useful for the validation of centroids from existing gene expression clustering algorithms. The neural gas (NG) clustering method according to [15] has been employed using Pearson correlation for centroid computation [16]. A number of 64 NG centroids has been embedded together with the gene expression data using HiT-MDS-2. The result displayed in Fig. 2 shows that the 64 centroids are well distributed among the embedded data, demonstrating that these clusters represent a continuum of data. Thereby, centroid numbers 1 to 8 and 63 to 64 depict similar expression patterns in neural gas clustering, which can be easily validated based on physical co-localization of centroid positions in the HiT-MDS-2 gene space plot (Fig. 2). Redundancy of the 64 centroids has been removed by summarizing them manually into the five shown major developmental patterns. These have been obtained by browsing and grouping temporally similar expressions, located at high-density peripheral regions of the bipolar embedding structure. From a global point of view, sets of coexpressed genes are identified reflecting the major cellular physiological events happening during endosperm development [see Additional file 2]. In conclusion, the output generated by HiT-MDS-2 provides faithful visualization of cluster relationships. This is a very helpful tool for the definition and validation of major centroids of gene expression profiles and for the assignment of their developmental patterns.

#### HiT-MDS-2 scatter plots for the visualization of relationships between functional categories of temporally coexpressed genes

In recent years, it has become general practice to subject high-throughput gene expression data to clustering methods and to browse the obtained clusters for finding representations of statistically significant functional categories of genes. Analysis by hierarchical clustering or k-means is usually complicated in the presence of high-dimensional input data and noisy outliers, the latter also affecting the interpretation of SOM clustering results. Statistical tests such as Fisher's exact test, ANOVA based global test, or gene set enrichment analysis (GSEA) produce useful hypotheses about significant transcriptional regulation [17], but they require that preconditions like certain data distributions are fulfilled and that test parameters are chosen carefully. Here, the neural gas clustering method is used with Pearson correlation similarity measure for computing cluster centroids. This method is known to yield consistent high-quality clusters, regardless of centroid initialization [15]. As with other centroid-based methods, though, the number of centroids required for deriving biological meaningful functional categorization can be hardly assessed in advance and induces additional data validation steps. By its correlation-preserving embedding facility, HiT-MDS-2 provides visual support of correlation structures and centroids by screening the spatial neighborhood of candidate genes to inspect whether they belong to clusters of certain functional categories. Here, we used manually annotated functional categories available for the 12 k barley seed array (N. Sreenivasulu and B. Usadel, unpublished data). The annotations are mapped to the embedding output of HiT-MDS-2 and get associated with corresponding expression profiles representing major developmental patterns of coexpressed genes. This mapping allows an easy transfer of biological information to the outcome of array experiments. Thereby, two levels of information are generated concerning (i) the identification of major pathways active in a particular stage of development, and (ii) the extraction of key regulators within transcriptionally co-regulated sets of genes.

**Figure 3:**
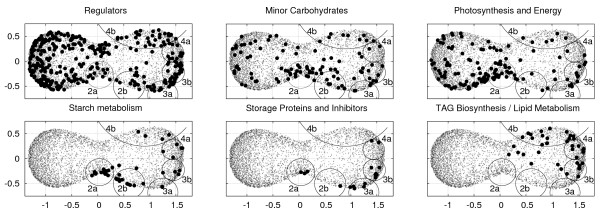
**Visual mapping of functional gene categories**. Mapping of six major functional categories to the HiT-MDS-2 scatter plot of genes. These categories can be directly related to the five prominent temporal patterns, cluster group 1–5, of gene expression in Fig. 2. Here, the focus is put on manually picked subspaces 2a & 2b of genes related to cluster group 2, 3a & 3b of group 3, and 4a & 4b of group 4. By browsing these subspaces defined by the encircled regions, key regulators can be identified that are closer to major genes of the storage pathway, storage proteins and inhibition, and TAG/lipid metabolism related genes. A list of coexpressed genes corresponding to the regions 2a/b, 3a/b, and 4a/b is provided extra [see Additional file 3]. Corresponding gene profiles are provided in a supplemental figure [see Additional file 6].

**(i) **The mapping of individual super-pathway information to the genome-wide graphical representation of the transcriptional response during plant ontogeny yields immediate hints about the occurrence of key biological processes during particular stages of development. For instance, this method, applied to transcriptome data of endosperm development, indicates that the abundance of genes related to photosynthesis, minor carbohydrates, and also for early steps of starch biosynthesis is characteristic of the intermediate stage (Fig. 3, cluster 2a) [see Additional file 3]. Clusters 2a-4b, described by the encircled regions, have been manually selected for focussing on (intermediate and storage) up regulation. These are related to the onset of storage events according to the down-stream pathway of starch metabolism (cluster 2b), storage proteins/protease inhibitors (cluster 3a and 3b) and TAG biosynthesis genes (cluster 3b, 4a and 4b). Such systematic activation of consecutive pathways reflects major physiological events happening in developing endosperm tissue. For instance, the end of the cell division phase is marked by an intermediate stage which is characteristic of the starch accumulation initiation. During this phase, coexpressed pathway genes are noticed that show tight physiological links to the photosynthesis-associated, ATP-producing energy metabolism, and to the production of carbon skeletons for synthesis of seed storage products. This initiation is followed by an accumulation of storage proteins at the peak of storage processes and lipid accumulation. As illustrated, such a mapping of functional information allows a serviceable transfer of biological knowledge to the outcome of array experiments.

**(ii) **Browsing the subspaces of the HiT-MDS-2 plot helped to identify key regulatory genes situated closer to major pathway genes, such as in case of starch, storage proteins, and oleosins. The highly correlated gene sets were extracted and compiled in a supplemental table [see Additional file 3]. As exemplary approach we discuss the coexpressed regulators of starch and storage protein transcripts in the following. The prominent transcription factors expressed during the intermediate development phase of endosperm tissue include 3 members of C3H/C3HC4, 2 chromatin remodeling factors, 1 bZIP, 1 ABI3/VP1, and 5 unclassified transcription factors. These are tightly coexpressed along with genes for photosynthesis, minor carbohydrates, as well as ADP-glucose pyrophosphorylase (AGPase) and sucrose synthase transcripts related to starch metabolism genes (cluster 2a) [see Additional file 3]. Among those regulators we noticed well-characterized regulatory factors, such as ABA response element binding factors (ABF3) from the bZIP family, and the abscisic acid insensitive protein 3 from the ABI3/VP1 family, of which its homologues are supposed to participate in promotion of reserve accumulation in dicots [18]. The correlation structure in the subspace of cluster 2b is related to expression of SNF1, bZIP transcription factor ABI5, MYB transcription factor, YABBY family transcription factor, Squamosa promoter binding factor, Auxin response factor and four unclassified transcription factors along with down-stream branching enzymes of starch metabolism and genes controlling minor carbohydrates [see Additional file 3].

As a main highlight, we observed expression of ABA response element binding factors (ABF3, ABI5 and ABI3) coexpressed along with SNF1 and starch biosynthetic genes during the intermediate stage and in the first storage peak of endosperm development. We found ABRE elements in the SNF1 kinase promoter region [12] which indicates a positive role of ABA in triggering these regulators. As recently demonstrated, ABA positively interacts with sugar signaling pathways in controlling key starch biosynthesis genes via SNF1 kinase [19]. Based on the correlative evidences, we also propose that SNF1 expression in endosperm is mediated by ABA via ABF3/ABI5, ABI3, which in turn might be responsible in regulating key genes of starch biosynthesis such as sucrose synthase and ADP-glucose pyrophosphorylase [12].

Another set of transcription factors, preferentially coexpressed along with transcripts of the hordein storage protein and the protease inhibitor during the main storage phase of endosperm development, includes 8 chromatin remodeling factors, 3 NAC, 2 DOF, and 9 unknown transcription factors. It was shown recently that two DOF transcription factors (SAD and BPBF) serve as activators of B1 storage protein genes during the maturation phase [20]. In the present study we also noticed (a) coexpression of two DOF family members, SAD and BPBF transcription factors along with hordein storage protein transcripts, and (b) in our recent study [12] we found enrichment of prolamin box cis-elements in upstream sequences of rice prolamin class storage protein genes (D, B1 and B3 hordeins). These evidences again point out that our detailed bioinformatics analysis of co-regulation of transcription does not only enhance our comprehensive knowledge of the developmental phenomena at gene regulation level, but it also helps to get initial glimpse of the systemic description of gene regulatory networks and their dynamics.

## Discussion

The validation of temporal gene expression centroids obtained by commonly used unsupervised clustering methods is a nontrivial task [4-6]. Since clustering results depend on the choice of method, the similarity measure, and the number of centroids, the assignment of expression profiles to clusters of interest does profit from faithful visual assistance. The proposed HiT-MDS-2 data embedding tool is designed to meet this purpose. Its versatile visualization abilities can be used to validate the results of centroid-based clustering methods, as has been demonstrated in the present study for the iterative neural gas clustering approach.

Moreover, HiT-MDS-2 scatter plots can be used for browsing interrelated temporal gene expression patterns (tightly coexpressed genes), and also the relationships between functional categories of coexpressed genes can be easily screened. Such a co-visualization of genes, exhibiting characteristic regulatory patterns, and their functional assignments is the major benefit of the nonlinear surrogate data representation realized by HiT-MDS-2.

An additional study has been carried out in order to demonstrate the generality of HiT-MDS-2 also for other data sets. We switched from the 12 k seed array containing EST clones selected from developing seed cDNA libraries (see results section) to 22 k Barley 1 Affymetrix chip in which oligos are compiled from at least 84 cDNA libraries encompassing various stages of plant ontogeny. This Affymetrix data set covers stages of developing endosperm tissue at 4, 8, 16 and 25 DAF in two replicate series. We applied two gene filtering criteria to the data set with (a) gene profiles with Pearson correlation greater 0.8 between the two available replicates and (b) at least 2-fold change between minimum and maximum expression values at 4, 8, 16 and 25 DAF. The filtered gene set contains 3031 differentially expressed high-quality genes. As shown in an additional figure, HiT-MDS-2 embedding of these genes produced a sandglass shape similar to Fig. 2 for 12 k seed data set [see Additional file 4]. Furthermore, clear global patterns of up-, down- and intermediate regulation are identified by browsing the obtained gene space [see Additional file 5]. This result confirms that the application of HiT-MDS-2 is not restricted to one specific data set but that it can be transfered to Affymetrix data as well. Thus, regulatory pattern structures revealed by HiT-MDS-2 are no artefacts of data selection, but they do reflect inherent properties of barley endosperm development.

### Comparison of Hit-MDS-2 with related visualization tools

**Figure 4:**
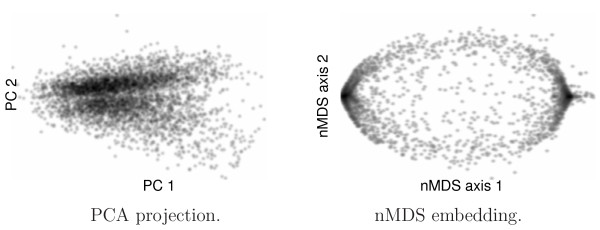
**Visualization of the gene space by other methods**. Density plots by PCA and nMDS, dark shading denoting high gene densities. For comparison, a much better and uniform visual spreading of the genes is provided by HiT-MDS-2, as displayed in Fig. 2.

Despite of the growing number of unsupervised clustering tools for gene expression data, currently only few visualization techniques offer intuitive validation of the clustering results. HiT-MDS-2 provides great flexibility in the choice of similarity measure, and also the dimensionality of the visualization can be chosen freely. One major advantage over SOM visualization is that the genes keep their individuality in the scatter display, which can be visually clustered on demand. Likewise, expression data and centroids from specific clustering methods can be embedded simultaneously for validation purposes. A standard data projection method like PCA puts too many constraints on the data similarity measure and on the modeling quality of surrogate data. By nature, PCA is restricted to the domain of Euclidean input spaces where variance is a properly defined concept [3]. Projection results of PCA are given in the left panel of Fig. 4. The density image displays the projection of the 4824 genes to the second principal component (PC2) against the projection to the first principal component (PC1). Two separated regions are revealed, the upper region corresponding to down-regulated gene profiles, the lower high-density region to up-regulated gene profiles. In contrast to correlation-based methods, the separation is not very strong, but the different structure of high-density regions indicates different regulatory characteristics specific to up-and down-regulation. The PCA result is complemented to the the much more advanced non-metric MDS (nMDS) method of Taguchi and Oono [8] shown in the right panel of Fig. 4. In comparison to PCA, many more details of the expression profile correlation structure is captured by the nMDS method. Like HiT-MDS-2 a bipolar structure appears, representing patterns of down-regulation at the left pole and up-regulation at the right pole. This density plot of nMDS is indeed very similar to the one obtained by HiT-MDS-2 for exponent *p *= 1 given in a supplemental figure [see Additional file 1]. However, since nMDS turns the implemented Pearson correlation input similarities (1 - **r**(**x**^*i*^, **x**^*j*^)) by a sorting operation into their ranks, there is no difference to the monotonic eighth power wrapper (1 - **r**(**x**^*i*^, **x**^*j*^))^8^. Compared to PCA and nMDS, the display of HiT-MDS-2 in Fig. 2, based on powers of correlation (*p *= 8), exhibits the characteristic bipolar sandglass shape representing not only magnified areas of up- and down-regulation, but also distinct intermediate regulation. A supplemental figure shows how the choice of exponent *p *can be used to emphasize specific correlation structure [see Additional file 1]. In principle, the XGvis system [11] is able to yield similar embedding results, but it requires that the similarity matrix is computed in advance as input to XGvis.

Regarding the computation efficiency, the HiT-MDS-2 algorithm is outstanding in the domain of metric MDS: it takes only 14 minutes and 21 seconds (861s) for 100 data cycles on a 3 GHz P4 processor for embedding the 4824 genes, while XGvis, for example, requires more than 4 hours for a comparable result. The nMDS approach, pursuing non-metric optimization, generated the displayed embedding within only 18 cycles using a relatively short time of 17 minutes and 21 seconds (1041s). Although, the PCA computation took less than 2 seconds on the reference PC with a 3 GHz Pentium 4 processor, the visualization cannot be used for screening temporal ordering gene expressions and is, hence, worthless for our purposes. Complementary to the visualization of the gene space, HiT-MDS-2 can also be used to display and evaluate hundreds of hybridized cDNA arrays without significant time requirement.

## Conclusion

HiT-MDS-2 allows creating faithful surrogate spaces, such as 2D scatter plots with Euclidean metric, from input spaces with custom data similarity measures. Fast convergence of the reconstructed space is obtained by stochastic optimization of an efficient correlation-based comparison of source and target space. For source data comparison, it has turned out that very useful graphical outputs are obtained when the short 14-dimensional expression time series of our 4824 genes are compared by 8th power of (1 - **r**(**x**^*i*^, **x**^*j*^)).

Resulting scatter plots of the well-distributed embedded points have been utilized in four ways: (1) for finding inter-sample correlations among experimental series; (2) for the detection of global regulatory gene expression patterns and for centroid validation; (3) for browsing the major temporal gene expression data and revealing the underlying functional pathway information; and (4) for visual mapping of regulatory genes co-localized with major functional gene categories. These features allow convenient visual screening of thousands of genes in parallel from time-course experiments. Although we have demonstrated only temporal data for screening co-responses in this study, HiT-MDS-2 can be also applied to highlight systematic differences among mutants or transgenics at multiple stages. The obtained visualizations help to get insights to massive data sets for approaching the goal of deriving new biological knowledge.

## Methods

Multi-dimensional scaling (MDS) implies the optimization of free parameters x^i∈X^n×d
 MathType@MTEF@5@5@+=feaafiart1ev1aaatCvAUfKttLearuWrP9MDH5MBPbIqV92AaeXatLxBI9gBaebbnrfifHhDYfgasaacH8akY=wiFfYdH8Gipec8Eeeu0xXdbba9frFj0=OqFfea0dXdd9vqai=hGuQ8kuc9pgc9s8qqaq=dirpe0xb9q8qiLsFr0=vr0=vr0dc8meaabaqaciaacaGaaeqabaqabeGadaaakeaaieqacuWF4baEgaqcamaaCaaaleqabaGaemyAaKgaaOGaeyicI4Saf8hwaGLbaKaadaWgaaWcbaGaemOBa4Maey41aqRaemizaqgabeaaaaa@378F@, i.e. locations of points x^i=(x^1i,...,x^di)
 MathType@MTEF@5@5@+=feaafiart1ev1aaatCvAUfKttLearuWrP9MDH5MBPbIqV92AaeXatLxBI9gBaebbnrfifHhDYfgasaacH8akY=wiFfYdH8Gipec8Eeeu0xXdbba9frFj0=OqFfea0dXdd9vqai=hGuQ8kuc9pgc9s8qqaq=dirpe0xb9q8qiLsFr0=vr0=vr0dc8meaabaqaciaacaGaaeqabaqabeGadaaakeaaieqacuWF4baEgaqcamaaCaaaleqabaGaemyAaKgaaOGaeyypa0JaeiikaGIafmiEaGNbaKaadaqhaaWcbaGaeGymaedabaGaemyAaKgaaOGaeiilaWIaeiOla4IaeiOla4IaeiOla4IaeiilaWIafmiEaGNbaKaadaqhaaWcbaGaemizaqgabaGaemyAaKgaaOGaeiykaKcaaa@3F68@ in a *d*-dimensional target space corresponding to *i *= 1...*n *input vectors **x**^*i *^∈ **X**_*n *× *q *_of dimension *q*. In case of the classical stress criterion, mutual distances d^ij=d(x^i,x^j)
 MathType@MTEF@5@5@+=feaafiart1ev1aaatCvAUfKttLearuWrP9MDH5MBPbIqV92AaeXatLxBI9gBaebbnrfifHhDYfgasaacH8akY=wiFfYdH8Gipec8Eeeu0xXdbba9frFj0=OqFfea0dXdd9vqai=hGuQ8kuc9pgc9s8qqaq=dirpe0xb9q8qiLsFr0=vr0=vr0dc8meaabaqaciaacaGaaeqabaqabeGadaaakeaaieaacuWFKbazgaqcamaaBaaaleaacqWGPbqAcqWGQbGAaeqaaOGaeyypa0Jae8hzaqMaeiikaGccbeGaf4hEaGNbaKaadaahaaWcbeqaaiabdMgaPbaakiabcYcaSiqb+Hha4zaajaWaaWbaaSqabeaacqWGQbGAaaGccqGGPaqkaaa@3C1D@ of all data pairs indexed by (*i, j*) should best fit the original distances d_*ij *_= d(**x**^*i*^, **x**^*j*^) in terms of the least squares s=∑i<jn(dij−d^ij)2=min⁡
 MathType@MTEF@5@5@+=feaafiart1ev1aaatCvAUfKttLearuWrP9MDH5MBPbIqV92AaeXatLxBI9gBaebbnrfifHhDYfgasaacH8akY=wiFfYdH8Gipec8Eeeu0xXdbba9frFj0=OqFfea0dXdd9vqai=hGuQ8kuc9pgc9s8qqaq=dirpe0xb9q8qiLsFr0=vr0=vr0dc8meaabaqaciaacaGaaeqabaqabeGadaaakeaaieaacqWFZbWCcqGH9aqpdaaeWaqaaiabcIcaOiab=rgaKnaaBaaaleaacqWGPbqAcqWGQbGAaeqaaaqaaiabdMgaPjabgYda8iabdQgaQbqaaiabd6gaUbqdcqGHris5aOGaeyOeI0Iaf8hzaqMbaKaadaWgaaWcbaGaemyAaKMaemOAaOgabeaakiabcMcaPmaaCaaaleqabaGaeGOmaidaaOGaeyypa0JagiyBa0MaeiyAaKMaeiOBa4gaaa@47B4@.

### Improvements of MDS (HiT-MDS-2)

A visual control, equivalent to least squares fit, is the Shepard diagram where on the d_*ij *_vs. d^ij
 MathType@MTEF@5@5@+=feaafiart1ev1aaatCvAUfKttLearuWrP9MDH5MBPbIqV92AaeXatLxBI9gBaebbnrfifHhDYfgasaacH8akY=wiFfYdH8Gipec8Eeeu0xXdbba9frFj0=OqFfea0dXdd9vqai=hGuQ8kuc9pgc9s8qqaq=dirpe0xb9q8qiLsFr0=vr0=vr0dc8meaabaqaciaacaGaaeqabaqabeGadaaakeaaieaacuWFKbazgaqcamaaBaaaleaacqWGPbqAcqWGQbGAaeqaaaaa@30F6@ plot all points should be located on the diagonal line of unit slope, i.e. (dij−d^ij)2=min⁡
 MathType@MTEF@5@5@+=feaafiart1ev1aaatCvAUfKttLearuWrP9MDH5MBPbIqV92AaeXatLxBI9gBaebbnrfifHhDYfgasaacH8akY=wiFfYdH8Gipec8Eeeu0xXdbba9frFj0=OqFfea0dXdd9vqai=hGuQ8kuc9pgc9s8qqaq=dirpe0xb9q8qiLsFr0=vr0=vr0dc8meaabaqaciaacaGaaeqabaqabeGadaaakeaacqGGOaakieaacqWFKbazdaWgaaWcbaGaemyAaKMaemOAaOgabeaakiabgkHiTiqb=rgaKzaajaWaaSbaaSqaaiabdMgaPjabdQgaQbqabaGccqGGPaqkdaahaaWcbeqaaiabikdaYaaakiabg2da9iGbc2gaTjabcMgaPjabc6gaUbaa@3E2B@. Although the Shepard plot is usually provided by MDS packages, it implies a misleadingly strict quality criterion: in most cases it is sufficient to maintain only the intra-distance relationships, while the scaling factor between source and target distances, i.e. the scale sizes of the corresponding point clouds, need not be unity. Thus, the strict least squares criterion can be relaxed to shift- and scale-invariant comparison by maximizing the Pearson correlation between the lower triangular source and target distance matrix:

rL(D,D^)=∑i<jn(dij−μD)⋅(d^ij−μD^)∑i<jn(dij−μD)2⋅∑i<jn(d^ij−μD^)2=:BC⋅D∈[−1;1]
 MathType@MTEF@5@5@+=feaafiart1ev1aaatCvAUfKttLearuWrP9MDH5MBPbIqV92AaeXatLxBI9gBaebbnrfifHhDYfgasaacH8akY=wiFfYdH8Gipec8Eeeu0xXdbba9frFj0=OqFfea0dXdd9vqai=hGuQ8kuc9pgc9s8qqaq=dirpe0xb9q8qiLsFr0=vr0=vr0dc8meaabaqaciaacaGaaeqabaqabeGadaaakqaaeeqaaGqabiab=jhaYnaaBaaaleaaieaacqGFmbataeqaaOGaeiikaGIae8hraqKaeiilaWIaf8hraqKbaKaacqGGPaqkcqGH9aqpdaWcaaqaamaaqadabaGaeiikaGIae4hzaq2aaSbaaSqaaiabdMgaPjabdQgaQbqabaGccqGHsisliiGacqqF8oqBdaWgaaWcbaGae8hraqeabeaakiabcMcaPiabgwSixlabcIcaOiqb+rgaKzaajaWaaSbaaSqaaiabdMgaPjabdQgaQbqabaGccqGHsislcqqF8oqBdaWgaaWcbaGaf8hraqKbaKaaaeqaaOGaeiykaKcaleaacqWGPbqAcqGH8aapcqWGQbGAaeaacqWGUbGBa0GaeyyeIuoaaOqaamaakaaabaWaaabmaeaacqGGOaakcqGFKbazdaWgaaWcbaGaemyAaKMaemOAaOgabeaakiabgkHiTiab9X7aTnaaBaaaleaacqWFebaraeqaaOGaeiykaKYaaWbaaSqabeaacqaIYaGmaaaabaGaemyAaKMaeyipaWJaemOAaOgabaGaemOBa4ganiabggHiLdaaleqaaOGaeyyXIC9aaOaaaeaadaaeWaqaaiabcIcaOiqb+rgaKzaajaWaaSbaaSqaaiabdMgaPjabdQgaQbqabaGccqGHsislcqqF8oqBdaWgaaWcbaGaf8hraqKbaKaaaeqaaOGaeiykaKYaaWbaaSqabeaacqaIYaGmaaaabaGaemyAaKMaeyipaWJaemOAaOgabaGaemOBa4ganiabggHiLdaaleqaaaaaaOqaaiabg2da9iabcQda6maalaaabaGaemOqaieabaWaaOaaaeaacqWGdbWqcqGHflY1cqWGebaraSqabaaaaOGaeyicI4Saei4waSLaeyOeI0IaeGymaeJaei4oaSJaeGymaeJaeiyxa0faaaa@88C4@

with μD˙=2n⋅(n−1)⋅∑i<jnd˙ij, D˙={D,D^}→d˙ij={dij,d^ij}.
 MathType@MTEF@5@5@+=feaafiart1ev1aaatCvAUfKttLearuWrP9MDH5MBPbIqV92AaeXatLxBI9gBaebbnrfifHhDYfgasaacH8akY=wiFfYdH8Gipec8Eeeu0xXdbba9frFj0=OqFfea0dXdd9vqai=hGuQ8kuc9pgc9s8qqaq=dirpe0xb9q8qiLsFr0=vr0=vr0dc8meaabaqaciaacaGaaeqabaqabeGadaaakeaaieaacqWF3bWDcqWFPbqAcqWF0baDcqWFObaAcqqGGaaiiiGacqGF8oqBdaWgaaWcbaacbeGaf0hraqKbaiaaaeqaaOGaeyypa0ZaaSaaaeaacqaIYaGmaeaacqWGUbGBcqGHflY1cqGGOaakcqWGUbGBcqGHsislcqaIXaqmcqGGPaqkaaGaeyyXIC9aaabCaeaacuWFKbazgaGaamaaBaaaleaacqWGPbqAcqWGQbGAaeqaaaqaaiabdMgaPjabgYda8iabdQgaQbqaaiabd6gaUbqdcqGHris5aOGaeiilaWIaeeiiaaIaf0hraqKbaiaacqGH9aqpcqGG7bWEcqqFebarcqGGSaalcuqFebargaqcaiabc2ha9jabgkziUkqb=rgaKzaacaWaaSbaaSqaaiabdMgaPjabdQgaQbqabaGccqGH9aqpcqGG7bWEcqWFKbazdaWgaaWcbaGaemyAaKMaemOAaOgabeaakiabcYcaSiqb=rgaKzaajaWaaSbaaSqaaiabdMgaPjabdQgaQbqabaGccqGG9bqFcqGGUaGlaaa@6CF1@

Matrix **D **= (d_*ij*_)_*i, j *= 1...*n *_contains pattern distances, and matrix D^=(d^ij)i,j=1...n
 MathType@MTEF@5@5@+=feaafiart1ev1aaatCvAUfKttLearuWrP9MDH5MBPbIqV92AaeXatLxBI9gBaebbnrfifHhDYfgasaacH8akY=wiFfYdH8Gipec8Eeeu0xXdbba9frFj0=OqFfea0dXdd9vqai=hGuQ8kuc9pgc9s8qqaq=dirpe0xb9q8qiLsFr0=vr0=vr0dc8meaabaqaciaacaGaaeqabaqabeGadaaakeaaieqacuWFebargaqcaiabg2da9iabcIcaOGqaaiqb+rgaKzaajaWaaSbaaSqaaiabdMgaPjabdQgaQbqabaGccqGGPaqkdaWgaaWcbaGaemyAaKMaeiilaWIaemOAaOMaeyypa0JaeGymaeJaeiOla4IaeiOla4IaeiOla4IaemOBa4gabeaaaaa@3EA9@ those of the reconstructions. In principle, input and output spaces are generic. However, the target configurations d^ij
 MathType@MTEF@5@5@+=feaafiart1ev1aaatCvAUfKttLearuWrP9MDH5MBPbIqV92AaeXatLxBI9gBaebbnrfifHhDYfgasaacH8akY=wiFfYdH8Gipec8Eeeu0xXdbba9frFj0=OqFfea0dXdd9vqai=hGuQ8kuc9pgc9s8qqaq=dirpe0xb9q8qiLsFr0=vr0=vr0dc8meaabaqaciaacaGaaeqabaqabeGadaaakeaaieaacuWFKbazgaqcamaaBaaaleaacqWGPbqAcqWGQbGAaeqaaaaa@30F6@ should be modeled by a Euclidean space for realizing intuitive low-dimensional spatial arrangements, such as 2D plots; furthermore, in the following, input distances d_*ij *_are expressed as dissimilarities by taking powers of gene profile correlations **r**. The two measures for reconstructions and input data are

d^ij=∑l=1d(x^li−x^lj)2, dij=(1−r(xi,xj))p.
 MathType@MTEF@5@5@+=feaafiart1ev1aaatCvAUfKttLearuWrP9MDH5MBPbIqV92AaeXatLxBI9gBaebbnrfifHhDYfgasaacH8akY=wiFfYdH8Gipec8Eeeu0xXdbba9frFj0=OqFfea0dXdd9vqai=hGuQ8kuc9pgc9s8qqaq=dirpe0xb9q8qiLsFr0=vr0=vr0dc8meaabaqaciaacaGaaeqabaqabeGadaaakeaafaqabeqacaaabaacbaGaf8hzaqMbaKaadaWgaaWcbaGaemyAaKMaemOAaOgabeaakiabg2da9maakaaabaWaaabmaeaacqGGOaakcuWG4baEgaqcamaaDaaaleaacqWGSbaBaeaacqWGPbqAaaGccqGHsislcuWG4baEgaqcamaaDaaaleaacqWGSbaBaeaacqWGQbGAaaGccqGGPaqkdaahaaWcbeqaaiabikdaYaaaaeaacqWGSbaBcqGH9aqpcqaIXaqmaeaacqWGKbaza0GaeyyeIuoaaSqabaGccqGGSaalaeaacqqGGaaicqWFKbazdaWgaaWcbaGaemyAaKMaemOAaOgabeaakiabg2da9maabmaabaGaeGymaeJaeyOeI0ccbeGae4NCaiNaeiikaGIae4hEaG3aaWbaaSqabeaacqWGPbqAaaGccqGGSaalcqGF4baEdaahaaWcbeqaaiabdQgaQbaakiabcMcaPaGaayjkaiaawMcaamaaCaaaleqabaGaemiCaahaaOGaeiOla4caaaaa@5C73@

Thereby integer exponents *p *≥ 1 control the discrimination of input data: in this study, a large value of *p *= 8 is used for separating clusters of highly correlated gene expression profiles, while *p *= 1 emphasizes the separation of anti-correlated patterns. The choice of the real value *p *> 0 is application-specific and up to the user's desire to accentuate the reconstruction of close or distant data.

In Eqn. 1 the abbreviated shorthand fraction is a literal one-to-one correspondence to the explicit term with sums. *B *= *B*(d^
 MathType@MTEF@5@5@+=feaafiart1ev1aaatCvAUfKttLearuWrP9MDH5MBPbIqV92AaeXatLxBI9gBaebbnrfifHhDYfgasaacH8akY=wiFfYdH8Gipec8Eeeu0xXdbba9frFj0=OqFfea0dXdd9vqai=hGuQ8kuc9pgc9s8qqaq=dirpe0xb9q8qiLsFr0=vr0=vr0dc8meaabaqaciaacaGaaeqabaqabeGadaaakeaaieaacuWFKbazgaqcaaaa@2E12@) is related to the mixed summation of both original and reconstructed distances, *D *= *D*(d^
 MathType@MTEF@5@5@+=feaafiart1ev1aaatCvAUfKttLearuWrP9MDH5MBPbIqV92AaeXatLxBI9gBaebbnrfifHhDYfgasaacH8akY=wiFfYdH8Gipec8Eeeu0xXdbba9frFj0=OqFfea0dXdd9vqai=hGuQ8kuc9pgc9s8qqaq=dirpe0xb9q8qiLsFr0=vr0=vr0dc8meaabaqaciaacaGaaeqabaqabeGadaaakeaaieaacuWFKbazgaqcaaaa@2E12@) refers to the dissimilarities dependent on the choices of the reconstructions X^
 MathType@MTEF@5@5@+=feaafiart1ev1aaatCvAUfKttLearuWrP9MDH5MBPbIqV92AaeXatLxBI9gBaebbnrfifHhDYfgasaacH8akY=wiFfYdH8Gipec8Eeeu0xXdbba9frFj0=OqFfea0dXdd9vqai=hGuQ8kuc9pgc9s8qqaq=dirpe0xb9q8qiLsFr0=vr0=vr0dc8meaabaqaciaacaGaaeqabaqabeGadaaakeaaieqacuWFybawgaqcaaaa@2DFB@, and *C *denotes the connection to the initially calculated and thus constant input pattern distances.

Instead of maximizing **r**_L _directly, minimization is performed on a very efficient stress function that inverts and stretches the domain [-1;1] of Pearson correlation for getting good convergence. In previous work, inverse power transformations of the correlation **r**_L _have been considered that worked reasonably well [9]. However, an exponent parameter required there had to be chosen carefully in combination with the step size of the stochastic gradient descent. Here, new formulas are derived for Fisher's Z' wrapper of the correlation **r**_L _given in Eqn. 1. This alternative transformation yields superior convergence while being more robust with respect to the choice of parameters.

The new stress function is based on Fisher's *negative *Z'-transformation:

s=−12⋅log⁡(a+rL(D,D^)a−rL(D,D^)),a=1+ε.
 MathType@MTEF@5@5@+=feaafiart1ev1aaatCvAUfKttLearuWrP9MDH5MBPbIqV92AaeXatLxBI9gBaebbnrfifHhDYfgasaacH8akY=wiFfYdH8Gipec8Eeeu0xXdbba9frFj0=OqFfea0dXdd9vqai=hGuQ8kuc9pgc9s8qqaq=dirpe0xb9q8qiLsFr0=vr0=vr0dc8meaabaqaciaacaGaaeqabaqabeGadaaakeaafaqabeqacaaabaacbaGae83CamNaeyypa0JaeyOeI0YaaSaaaeaacqaIXaqmaeaacqaIYaGmaaGaeyyXICTagiiBaWMaei4Ba8Maei4zaC2aaeWaaeaadaWcaaqaaiabdggaHjabgUcaRGqabiab+jhaYnaaBaaaleaacqWFmbataeqaaOGaeiikaGIae4hraqKaeiilaWIaf4hraqKbaKaacqGGPaqkaeaacqWGHbqycqGHsislcqGFYbGCdaWgaaWcbaGae8htaWeabeaakiabcIcaOiab+reaejabcYcaSiqb+reaezaajaGaeiykaKcaaaGaayjkaiaawMcaaiabcYcaSaqaaiabdggaHjabg2da9iabigdaXiabgUcaRGGaciab9v7aLbaacqGGUaGlaaa@54FF@

Fisher's original formulation implies *a *= 1; here, however, potential singularities are prevented by *a *> 1.

The stress function *s *is minimized by optimally arranging the reconstruction points X^
 MathType@MTEF@5@5@+=feaafiart1ev1aaatCvAUfKttLearuWrP9MDH5MBPbIqV92AaeXatLxBI9gBaebbnrfifHhDYfgasaacH8akY=wiFfYdH8Gipec8Eeeu0xXdbba9frFj0=OqFfea0dXdd9vqai=hGuQ8kuc9pgc9s8qqaq=dirpe0xb9q8qiLsFr0=vr0=vr0dc8meaabaqaciaacaGaaeqabaqabeGadaaakeaaieqacuWFybawgaqcaaaa@2DFB@ in the Euclidean target space. This is achieved by a gradient descent on the stress function s, which requires finding zeros of the derivatives of s with respect to the free parameters x^ki
 MathType@MTEF@5@5@+=feaafiart1ev1aaatCvAUfKttLearuWrP9MDH5MBPbIqV92AaeXatLxBI9gBaebbnrfifHhDYfgasaacH8akY=wiFfYdH8Gipec8Eeeu0xXdbba9frFj0=OqFfea0dXdd9vqai=hGuQ8kuc9pgc9s8qqaq=dirpe0xb9q8qiLsFr0=vr0=vr0dc8meaabaqaciaacaGaaeqabaqabeGadaaakeaacuWG4baEgaqcamaaDaaaleaacqWGRbWAaeaacqWGPbqAaaaaaa@311C@:

s=−Z′∘rL∘D^∘X^→min⁡
 MathType@MTEF@5@5@+=feaafiart1ev1aaatCvAUfKttLearuWrP9MDH5MBPbIqV92AaeXatLxBI9gBaebbnrfifHhDYfgasaacH8akY=wiFfYdH8Gipec8Eeeu0xXdbba9frFj0=OqFfea0dXdd9vqai=hGuQ8kuc9pgc9s8qqaq=dirpe0xb9q8qiLsFr0=vr0=vr0dc8meaabaqaciaacaGaaeqabaqabeGadaaakeaaieaacqWFZbWCcqGH9aqpcqGHsislcuWGAbGwgaqbaiablIHiVHqabiab+jhaYnaaBaaaleaacqWFmbataeqaaOGaeSigI8Maf4hraqKbaKaacqWIyiYBcuGFybawgaqcaiabgkziUkGbc2gaTjabcMgaPjabc6gaUbaa@403E@

⇒∂s∂x^ki=−∑j=1...nj≠i∂Z′∂rL⋅∂rL∂d^ij⋅∂d^ij∂x^ki→0, i=1...n
 MathType@MTEF@5@5@+=feaafiart1ev1aaatCvAUfKttLearuWrP9MDH5MBPbIqV92AaeXatLxBI9gBaebbnrfifHhDYfgasaacH8akY=wiFfYdH8Gipec8Eeeu0xXdbba9frFj0=OqFfea0dXdd9vqai=hGuQ8kuc9pgc9s8qqaq=dirpe0xb9q8qiLsFr0=vr0=vr0dc8meaabaqaciaacaGaaeqabaqabeGadaaakeaacqGHshI3daWcaaqaaGGaciab=jGi2Iqaaiab+nhaZbqaaiab=jGi2kqbdIha4zaajaWaa0baaSqaaiabdUgaRbqaaiabdMgaPbaaaaGccqGH9aqpcqGHsisldaaeWbqaamaalaaabaGae8NaIyRafmOwaOLbauaaaeaacqWFciITieqacqqFYbGCdaWgaaWcbaGae4htaWeabeaaaaGccqGHflY1daWcaaqaaiab=jGi2kab9jhaYnaaBaaaleaacqGFmbataeqaaaGcbaGae8NaIyRaf4hzaqMbaKaadaWgaaWcbaGaemyAaKMaemOAaOgabeaaaaGccqGHflY1daWcaaqaaiab=jGi2kqb+rgaKzaajaWaaSbaaSqaaiabdMgaPjabdQgaQbqabaaakeaacqWFciITcuWG4baEgaqcamaaDaaaleaacqWGRbWAaeaacqWGPbqAaaaaaaqaaiabdQgaQjabg2da9iabigdaXiabc6caUiabc6caUiabc6caUiabd6gaUbqaaiabdQgaQjabgcMi5kabdMgaPbqdcqGHris5aOGaeyOKH4QaeGimaaJaeiilaWIaeeiiaaIaemyAaKMaeyypa0JaeGymaeJaeiOla4IaeiOla4IaeiOla4IaemOBa4gaaa@7492@

Solutions are found by iterative updates of randomly drawn points *i *by Δx^ki=−γ⋅∂s∂x^ki
 MathType@MTEF@5@5@+=feaafiart1ev1aaatCvAUfKttLearuWrP9MDH5MBPbIqV92AaeXatLxBI9gBaebbnrfifHhDYfgasaacH8akY=wiFfYdH8Gipec8Eeeu0xXdbba9frFj0=OqFfea0dXdd9vqai=hGuQ8kuc9pgc9s8qqaq=dirpe0xb9q8qiLsFr0=vr0=vr0dc8meaabaqaciaacaGaaeqabaqabeGadaaakeaacqGHuoarcuWG4baEgaqcamaaDaaaleaacqWGRbWAaeaacqWGPbqAaaGccqGH9aqpcqGHsisliiGacqWFZoWzcqGHflY1daWcaaqaaiab=jGi2Iqaaiab+nhaZbqaaiab=jGi2kqbdIha4zaajaWaa0baaSqaaiabdUgaRbqaaiabdMgaPbaaaaaaaa@4129@ of step size *γ *into the direction of the steepest gradient of s. Although convergence to a global optimum cannot be claimed by such an approach, final point configurations have been found to be very stable and of high quality in different runs. The required derivatives of Eqn. 4 are

∂Z′∂rL=aa2−rL2∂rL∂d^ij=(dij−μD)⋅D−(d^ij−μD^)⋅BD⋅C⋅D∂d^ij∂x^ki=(x^ki−x^kj)/d^ij.
 MathType@MTEF@5@5@+=feaafiart1ev1aaatCvAUfKttLearuWrP9MDH5MBPbIqV92AaeXatLxBI9gBaebbnrfifHhDYfgasaacH8akY=wiFfYdH8Gipec8Eeeu0xXdbba9frFj0=OqFfea0dXdd9vqai=hGuQ8kuc9pgc9s8qqaq=dirpe0xb9q8qiLsFr0=vr0=vr0dc8meaabaqaciaacaGaaeqabaqabeGadaaakqaaeeqaamaalaaabaacciGae8NaIyRafmOwaOLbauaaaeaacqWFciITieqacqGFYbGCdaWgaaWcbaacbaGae0htaWeabeaaaaGccqGH9aqpdaWcaaqaaiabdggaHbqaaiabdggaHnaaCaaaleqabaGaeGOmaidaaOGaeyOeI0Iae4NCai3aa0baaSqaaiabbYeambqaaiabbkdaYaaaaaaakeaadaWcaaqaaiab=jGi2kab+jhaYnaaBaaaleaacqqFmbataeqaaaGcbaGae8NaIyRaf0hzaqMbaKaadaWgaaWcbaGaemyAaKMaemOAaOgabeaaaaGccqGH9aqpdaWcaaqaaiabcIcaOiab9rgaKnaaBaaaleaacqWGPbqAcqWGQbGAaeqaaOGaeyOeI0Iae8hVd02aaSbaaSqaaiab+reaebqabaGccqGGPaqkcqGHflY1ieGacqaFebarcqGHsislcqGGOaakcuqFKbazgaqcamaaBaaaleaacqWGPbqAcqWGQbGAaeqaaOGaeyOeI0Iae8hVd02aaSbaaSqaaiqb+reaezaajaaabeaakiabcMcaPiabgwSixlabdkeacbqaaiabdseaejabgwSixpaakaaabaGaem4qamKaeyyXICTaemiraqealeqaaaaaaOqaamaalaaabaGae8NaIyRaf0hzaqMbaKaadaWgaaWcbaGaemyAaKMaemOAaOgabeaaaOqaaiab=jGi2kqbdIha4zaajaWaa0baaSqaaiabdUgaRbqaaiabdMgaPbaaaaGccqGH9aqpcqGGOaakcuWG4baEgaqcamaaDaaaleaacqWGRbWAaeaacqWGPbqAaaGccqGHsislcuWG4baEgaqcamaaDaaaleaacqWGRbWAaeaacqWGQbGAaaGccqGGPaqkcqGGVaWlcuqFKbazgaqcamaaBaaaleaacqWGPbqAcqWGQbGAaeqaaOGaeiOla4caaaa@8979@

The two parameters of the new HiT-MDS-2 are non-critical and they can be fixed to *γ *= 0.1 and *a *= 1.001 in most cases. This robustness is a substantial advantage over the first HiT-MDS formulation described in [9], where a tight coupling of an additional parameter with the learning rate *γ *required three to five re-runs of the algorithm with appropriately chosen parameters. As a consequence, the old version took on average four times longer to converge to the same final results like the new HiT-MDS-2.

The embedding procedure is outlined in Algorithm 1. Initially, a random projection of the high-dimensional source data is calculated and the resulting similarity matrix is correlated with the mean-subtracted original one for obtaining *B, C *and *D*. Mean subtraction from **D **does not affect the Pearson correlation **r**_L_(**D**, D^
 MathType@MTEF@5@5@+=feaafiart1ev1aaatCvAUfKttLearuWrP9MDH5MBPbIqV92AaeXatLxBI9gBaebbnrfifHhDYfgasaacH8akY=wiFfYdH8Gipec8Eeeu0xXdbba9frFj0=OqFfea0dXdd9vqai=hGuQ8kuc9pgc9s8qqaq=dirpe0xb9q8qiLsFr0=vr0=vr0dc8meaabaqaciaacaGaaeqabaqabeGadaaakeaaieqacuWFebargaqcaaaa@2DD3@), but simplifies further calculations. More substantial speed-up is obtained by exploiting the symmetry of **D **and D^
 MathType@MTEF@5@5@+=feaafiart1ev1aaatCvAUfKttLearuWrP9MDH5MBPbIqV92AaeXatLxBI9gBaebbnrfifHhDYfgasaacH8akY=wiFfYdH8Gipec8Eeeu0xXdbba9frFj0=OqFfea0dXdd9vqai=hGuQ8kuc9pgc9s8qqaq=dirpe0xb9q8qiLsFr0=vr0=vr0dc8meaabaqaciaacaGaaeqabaqabeGadaaakeaaieqacuWFebargaqcaaaa@2DD3@ and by implementing differential updates of *B *and *D *corresponding to changes in single rows and columns of D^
 MathType@MTEF@5@5@+=feaafiart1ev1aaatCvAUfKttLearuWrP9MDH5MBPbIqV92AaeXatLxBI9gBaebbnrfifHhDYfgasaacH8akY=wiFfYdH8Gipec8Eeeu0xXdbba9frFj0=OqFfea0dXdd9vqai=hGuQ8kuc9pgc9s8qqaq=dirpe0xb9q8qiLsFr0=vr0=vr0dc8meaabaqaciaacaGaaeqabaqabeGadaaakeaaieqacuWFebargaqcaaaa@2DD3@ during the iterative embedding process: further details on an efficient realization of line 12 in Algorithm 1 are given in [9]. Finally, stochastic gradient descent on s maximizes the correlation iteratively by moving the target points x^i
 MathType@MTEF@5@5@+=feaafiart1ev1aaatCvAUfKttLearuWrP9MDH5MBPbIqV92AaeXatLxBI9gBaebbnrfifHhDYfgasaacH8akY=wiFfYdH8Gipec8Eeeu0xXdbba9frFj0=OqFfea0dXdd9vqai=hGuQ8kuc9pgc9s8qqaq=dirpe0xb9q8qiLsFr0=vr0=vr0dc8meaabaqaciaacaGaaeqabaqabeGadaaakeaaieqacuWF4baEgaqcamaaCaaaleqabaGaemyAaKgaaaaa@2FC3@ into proper places until a saturated quality level is reached.

#### Features of HiT-MDS-2

Embedded point distances maximize correlation with source data similarities for a faithful display of relationships. Classical MDS stress applied to Euclidean data yields final configurations equivalent to the PCA projection [21]. The HiT-MDS-2 criterion, though, provides more degrees of freedom and allows thus fast convergence and improved displays. Any symmetric similarity matrix of relationships between input data can be processed, but different powers of correlation measures turn out to be preferable in the context of gene expression mining. In principle, for Euclidean target displays, MDS axes of embedded data do not carry any special meaning, because the embedding procedure is invariant to offsets, scaling, sign-flipping, and rotation; thus, there is no preferred intrinsic direction. What counts is the arrangement of inter-point distances only. Final displays can and should be normalized

1: Read input data **X**.

2: Initialize X^
 MathType@MTEF@5@5@+=feaafiart1ev1aaatCvAUfKttLearuWrP9MDH5MBPbIqV92AaeXatLxBI9gBaebbnrfifHhDYfgasaacH8akY=wiFfYdH8Gipec8Eeeu0xXdbba9frFj0=OqFfea0dXdd9vqai=hGuQ8kuc9pgc9s8qqaq=dirpe0xb9q8qiLsFr0=vr0=vr0dc8meaabaqaciaacaGaaeqabaqabeGadaaakeaaieqacuWFybawgaqcaaaa@2DFB@ by random projection X^n×d=Xn×q⋅Rq×d
 MathType@MTEF@5@5@+=feaafiart1ev1aaatCvAUfKttLearuWrP9MDH5MBPbIqV92AaeXatLxBI9gBaebbnrfifHhDYfgasaacH8akY=wiFfYdH8Gipec8Eeeu0xXdbba9frFj0=OqFfea0dXdd9vqai=hGuQ8kuc9pgc9s8qqaq=dirpe0xb9q8qiLsFr0=vr0=vr0dc8meaabaqaciaacaGaaeqabaqabeGadaaakeaaieqacuWFybawgaqcamaaBaaaleaacqWGUbGBcqGHxdaTcqWGKbazaeqaaOGaeyypa0Jae8hwaG1aaSbaaSqaaiabd6gaUjabgEna0kabdghaXbqabaGccqGHflY1ieWacqGFsbGudaWgaaWcbaGaemyCaeNaey41aqRaemizaqgabeaaaaa@42D3@.

3: Calculate input matrix **D **and subtract mean ⇒ constant *C*.

4: Calculate target distances D^
 MathType@MTEF@5@5@+=feaafiart1ev1aaatCvAUfKttLearuWrP9MDH5MBPbIqV92AaeXatLxBI9gBaebbnrfifHhDYfgasaacH8akY=wiFfYdH8Gipec8Eeeu0xXdbba9frFj0=OqFfea0dXdd9vqai=hGuQ8kuc9pgc9s8qqaq=dirpe0xb9q8qiLsFr0=vr0=vr0dc8meaabaqaciaacaGaaeqabaqabeGadaaakeaaieqacuWFebargaqcaaaa@2DD3@ ⇒ initial *B, D*.

5: **repeat**

6:   Draw a pattern index 1 ≤ *i *≤ *n *from randomly shuffled list.

7:   **for all ***j *≠ *i *do

8:      Δx^i←Δx^i−∂s∂x^ki
 MathType@MTEF@5@5@+=feaafiart1ev1aaatCvAUfKttLearuWrP9MDH5MBPbIqV92AaeXatLxBI9gBaebbnrfifHhDYfgasaacH8akY=wiFfYdH8Gipec8Eeeu0xXdbba9frFj0=OqFfea0dXdd9vqai=hGuQ8kuc9pgc9s8qqaq=dirpe0xb9q8qiLsFr0=vr0=vr0dc8meaabaqaciaacaGaaeqabaqabeGadaaakeaacqGHuoarieqacuWF4baEgaqcamaaCaaaleqabaGaemyAaKgaaOGaeyiKHWQaeyiLdqKaf8hEaGNbaKaadaahaaWcbeqaaiabdMgaPbaakiabgkHiTmaalaaabaacciGae4NaIylcbaGae03CamhabaGae4NaIyRafmiEaGNbaKaadaqhaaWcbaGaem4AaSgabaGaemyAaKgaaaaaaaa@4142@ { accumulate derivatives (Eqn. 4) }

9:   **end for**

10:   x^i←x^i+γ⋅Δx^i
 MathType@MTEF@5@5@+=feaafiart1ev1aaatCvAUfKttLearuWrP9MDH5MBPbIqV92AaeXatLxBI9gBaebbnrfifHhDYfgasaacH8akY=wiFfYdH8Gipec8Eeeu0xXdbba9frFj0=OqFfea0dXdd9vqai=hGuQ8kuc9pgc9s8qqaq=dirpe0xb9q8qiLsFr0=vr0=vr0dc8meaabaqaciaacaGaaeqabaqabeGadaaakeaaieqacuWF4baEgaqcamaaCaaaleqabaGaemyAaKgaaOGaeyiKHWQaf8hEaGNbaKaadaahaaWcbeqaaiabdMgaPbaakiabgUcaRGGaciab+n7aNjabgwSixlabgs5aejqb=Hha4zaajaWaaWbaaSqabeaacqWGPbqAaaaaaa@3E1A@ { adapt location of target point }

11:   Recalculate distances d^(x^i,x^j)
 MathType@MTEF@5@5@+=feaafiart1ev1aaatCvAUfKttLearuWrP9MDH5MBPbIqV92AaeXatLxBI9gBaebbnrfifHhDYfgasaacH8akY=wiFfYdH8Gipec8Eeeu0xXdbba9frFj0=OqFfea0dXdd9vqai=hGuQ8kuc9pgc9s8qqaq=dirpe0xb9q8qiLsFr0=vr0=vr0dc8meaabaqaciaacaGaaeqabaqabeGadaaakeaaieaacuWFKbazgaqcaiabcIcaOGqabiqb+Hha4zaajaWaaWbaaSqabeaacqWGPbqAaaGccqGGSaalcuGF4baEgaqcamaaCaaaleqabaGaemOAaOgaaOGaeiykaKcaaa@36DC@ influenced by new point x^i
 MathType@MTEF@5@5@+=feaafiart1ev1aaatCvAUfKttLearuWrP9MDH5MBPbIqV92AaeXatLxBI9gBaebbnrfifHhDYfgasaacH8akY=wiFfYdH8Gipec8Eeeu0xXdbba9frFj0=OqFfea0dXdd9vqai=hGuQ8kuc9pgc9s8qqaq=dirpe0xb9q8qiLsFr0=vr0=vr0dc8meaabaqaciaacaGaaeqabaqabeGadaaakeaaieqacuWF4baEgaqcamaaCaaaleqabaGaemyAaKgaaaaa@2FC3@;

12:      thereby, update differential changes in *B *and *D*.

13: **until **convergence criterion is met.

14: Postprocess: center X^
 MathType@MTEF@5@5@+=feaafiart1ev1aaatCvAUfKttLearuWrP9MDH5MBPbIqV92AaeXatLxBI9gBaebbnrfifHhDYfgasaacH8akY=wiFfYdH8Gipec8Eeeu0xXdbba9frFj0=OqFfea0dXdd9vqai=hGuQ8kuc9pgc9s8qqaq=dirpe0xb9q8qiLsFr0=vr0=vr0dc8meaabaqaciaacaGaaeqabaqabeGadaaakeaaieqacuWFybawgaqcaaaa@2DFB@, normalize by largest dimension variance.

15: Optional coordinate rotation: project X^
 MathType@MTEF@5@5@+=feaafiart1ev1aaatCvAUfKttLearuWrP9MDH5MBPbIqV92AaeXatLxBI9gBaebbnrfifHhDYfgasaacH8akY=wiFfYdH8Gipec8Eeeu0xXdbba9frFj0=OqFfea0dXdd9vqai=hGuQ8kuc9pgc9s8qqaq=dirpe0xb9q8qiLsFr0=vr0=vr0dc8meaabaqaciaacaGaaeqabaqabeGadaaakeaaieqacuWFybawgaqcaaaa@2DFB@ to eigenvectors.

Algorithm 1: HiT-MDS-2

for comparison purposes. Thereby, four steps of (1) mean centering, (2) variance-based rescaling, (3) PCA coordinate rotation, and (4) skewness-based sign flipping properly resolve embedding invariances while maintaining the reconstructed distance relationships. Furthermore, density plots of the embedded data can be computed by using symmetric Gaussian kernels in order to inspect the similarity densities in the data space. GNU Octave/MATLAB and R implementations as well as fast C source code of the HiT-MDS-2 algorithm are available under GPLv2 license [22].

#### Data preparation

A total of 330 008 gene expression values collected from 28 hybridization experiments with 12 k macroarrays, covering 14 temporal developmental points from two independent series, were considered for data processing. As a first quality criterion, gene expression values surpassing twice the background level are considered. Background subtraction is carried out for the remaining genes, followed by quantile normalization. This processing is done separately for each series to allow the comparison of signal intensities across time series. As usual, log_2_-transformed final expression values are considered. Cubic spline smoothing with moderate smoothing parameter has been applied to each temporal gene expression profile. A filter based on Pearson similarity has been applied to select gene profile time series that correlate at a conservative level of *r *> 0.5 between the two independent series. With this criterion, a qualified subset of 4824 out of 11786 genes has been created for analysis.

## Authors' contributions

MS implemented the software and applied it to gene expression data. MS and NS designed the study and prepared the manuscript. BU and NS contributed the barley ontologies with functional categories. All authors have read and approved the final manuscript.

## Supplementary Material

Additional file 1 Additional HiT-MDS-2 embeddings of the expression data set containing 4824 genes, using different exponents p. Different exponents used in the data similarity measure (1 - **r**(**x**^*i*^, **x**^*j*^))^*p *^highlight specific correlation structures in the corresponding HiT-MDS-2 embeddings. Results for exponents *p *= 24, 4,1,0.25 are shown in panels a-d, respectively. Click here for file

Additional file 2 List of 4824 genes with 64 centroids. The table contains gene expressions of 4824 high-quality genes covering 14 developmental stages, ODAF-26DAF, in steps of two days. Expression levels are log_2_-transformed quantile-normalized values. Genes are assigned to 64 centroids from neural gas clustering. Click here for file

Additional file 3 Table of functional categories. The table contains genes with manually assigned major functional categories corresponding to Fig. 3 of the manuscript. Click here for file

Additional file 4 HiT-MDS-2 embedding of gene expressions of 3031 filtered genes from developing barley endosperm at time points 4, 8, 16, 25 days after flowering. Expression levels are taken from Barley 1 Affymetrix chip. Like in Figure 2 of the manuscript, a sandglass shape is obtained for a correlation exponent of p = 8. Since only four time points are considered, the four-dimensional expression vectors are very faithfully represented in the scatter plot. The corresponding regulation patterns of up-, down- and intermediate regulation are displayed in an extra figure [see Additional file 5]. Click here for file

Additional file 5 Gene expression profiles obtained from Barley 1 Affymetrix gene chip connected to highlighted regions of the HiT-MDS-2 gene space plot [see Additional file 4]. High spatial specificity is observed in the exemplary clusters 1–8 covering interesting locations in the gene space. Patterns of up- and down-regulation fall into opposite poles. Cluster number 5 shows intermediate up-regulation with quite diverse characteristic, where Pearson correlation does not yield good discrimination between peaks in the second or third temporal stage. Cluster number 3 contains genes that become active just at the last stage (day 25 after flowering). Click here for file

Additional file 6 Gene expression profiles corresponding to the six sub-clusters 2a, 2b, 3a, 3b, 4a and 4b referred in Figure 3 of the manuscript. The expression profiles reflect z-score normalized log_2 _values. In addition to individual gene expression curves displayed in blue, their mean and standard deviation are depicted by red lines. Highest variability is observed for intermediate regulation events in cluster 2a; yet, the overall quality of coexpression in the six clusters is represented well. Click here for file
