# Supplementary material for: Correlation-maximizing surrogate gene space for visual mining of gene expression patterns in developing barley endosperm tissue
Source: BMC Bioinformatics. 2007 May 22;8:165. doi: 10.1186/1471-2105-8-165 (PMC1891114; doi:10.1186/1471-2105-8-165)

Supplemental figure S1

Additional HiT-MDS-2 embeddings of gene expressions for different exponents p.

(a) p=24

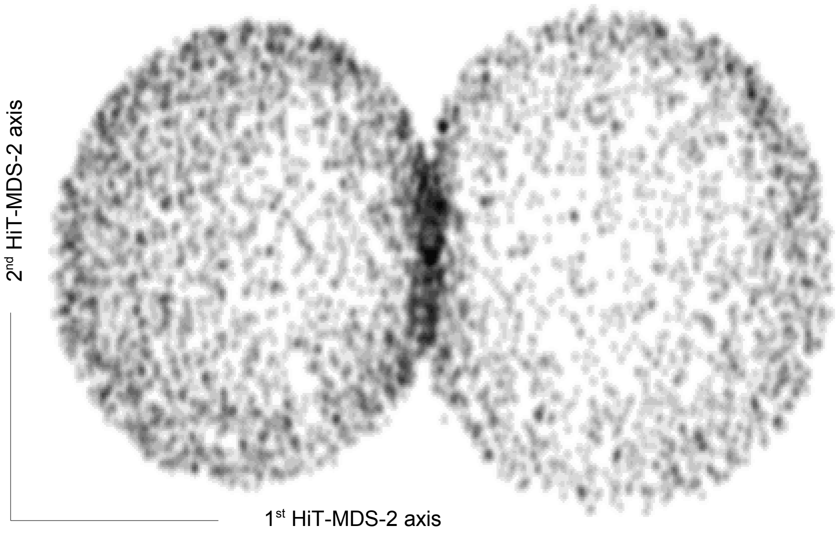

(b) p=4

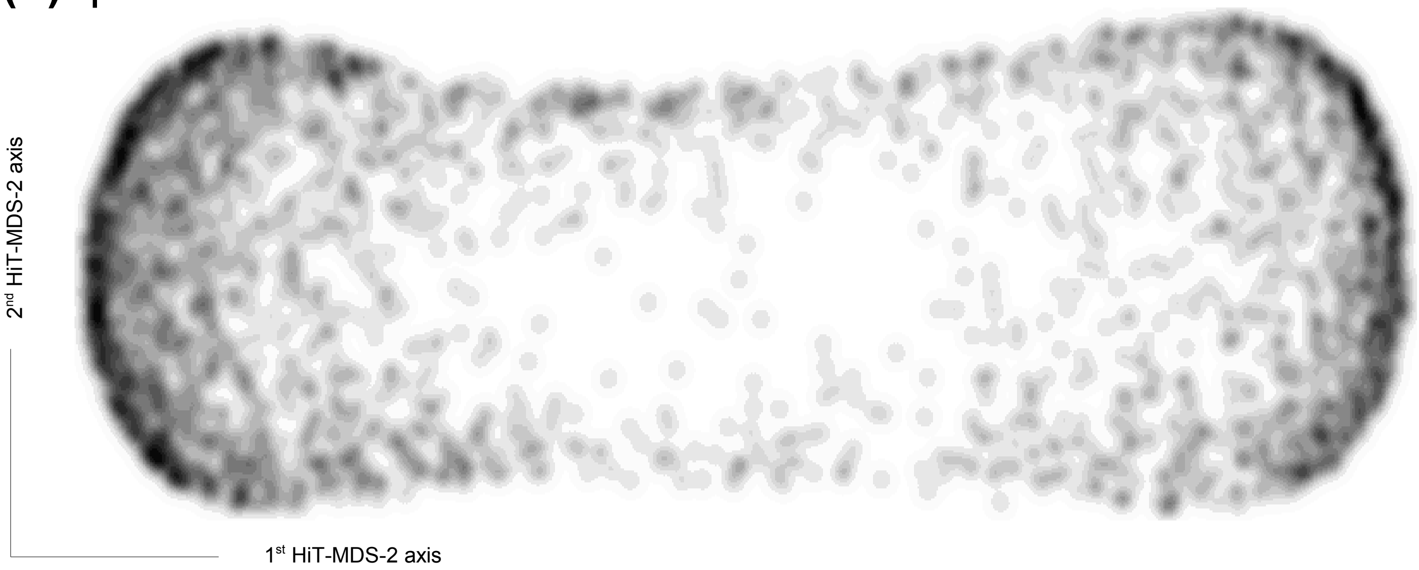

(c) p=1

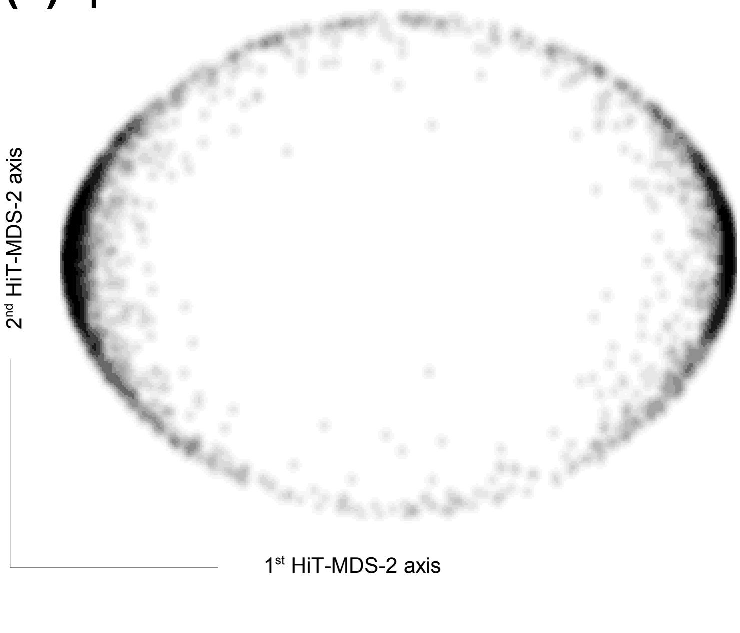

(d) p=0.25

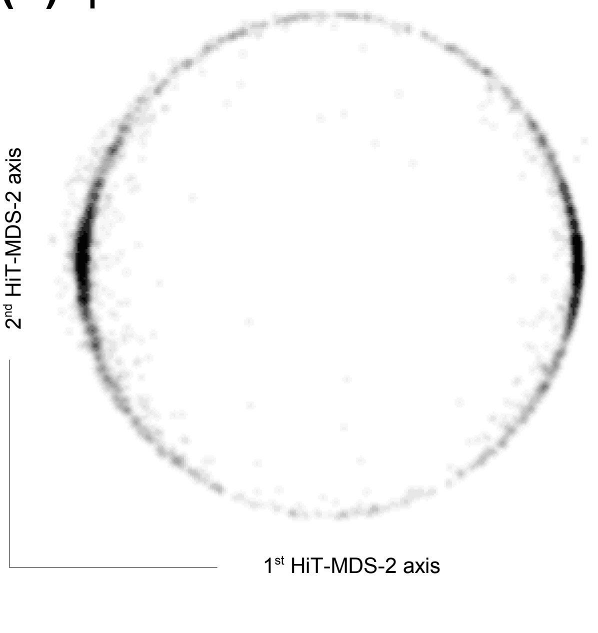

Supplement: Additional file 1 — Additional HiT-MDS-2 embeddings of the expression data set containing 4824 genes, using different exponents p. Different exponents used in the data similarity measure (1 - r(xi, xj))p highlight specific correlation structures in the corresponding HiT-MDS-2 embeddings. Results for exponents p = 24, 4,1,0.25 are shown in panels a-d, respectively. [file 1471-2105-8-165-S1.pdf]
