# Supplementary material for: Correlation-maximizing surrogate gene space for visual mining of gene expression patterns in developing barley endosperm tissue
Source: BMC Bioinformatics. 2007 May 22;8:165. doi: 10.1186/1471-2105-8-165 (PMC1891114; doi:10.1186/1471-2105-8-165)
Supplement: Additional file 4 — HiT-MDS-2 embedding of gene expressions of 3031 filtered genes from developing barley endosperm at time points 4, 8, 16, 25 days after flowering. Expression levels are taken from Barley 1 Affymetrix chip. Like in Figure 2 of the manuscript, a sandglass shape is obtained for a correlation exponent of p = 8. Since only four time points are considered, the four-dimensional expression vectors are very faithfully represented in the scatter plot. The corresponding regulation patterns of up-, down- and intermediate regulation are displayed in an extra figure [see Additional file 5]. [file 1471-2105-8-165-S4.pdf]

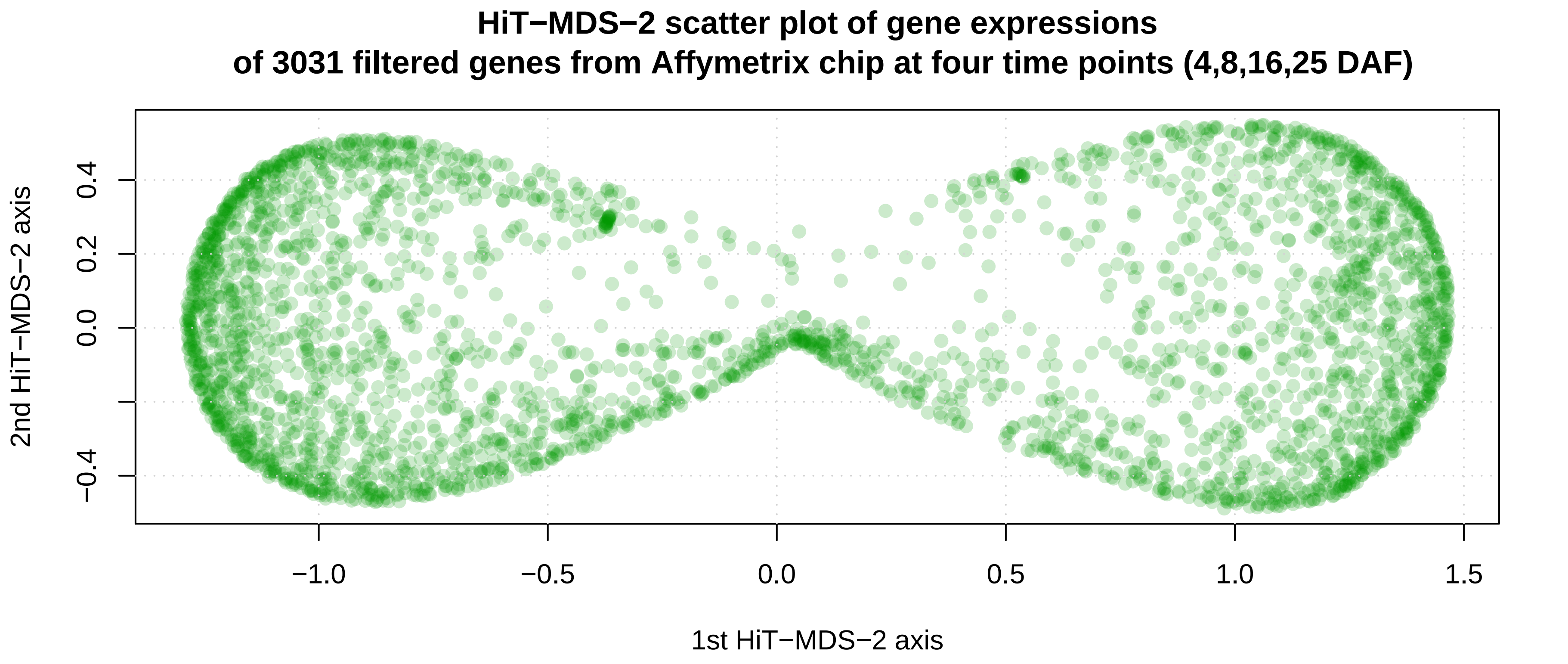

HiT−MDS−2 scatter plot of gene expressions
of 3031 filtered genes from Affymetrix chip at four time points (4,8,16,25 DAF)
2nd HiT−MDS−2 axis
−0.4
0.0
0.2
0.4
−1.0
−0.5
0.0
0.5
1.0
1.5
1st HiT−MDS−2 axis
