# Supplementary figures and images for: Correlation-maximizing surrogate gene space for visual mining of gene expression patterns in developing barley endosperm tissue
Source: BMC Bioinformatics. 2007 May 22;8:165. doi: 10.1186/1471-2105-8-165 (PMC1891114; doi:10.1186/1471-2105-8-165)

2<sup>nd</sup> HiT-MDS-2 axis

1<sup>st</sup> HiT-MDS-2 axis

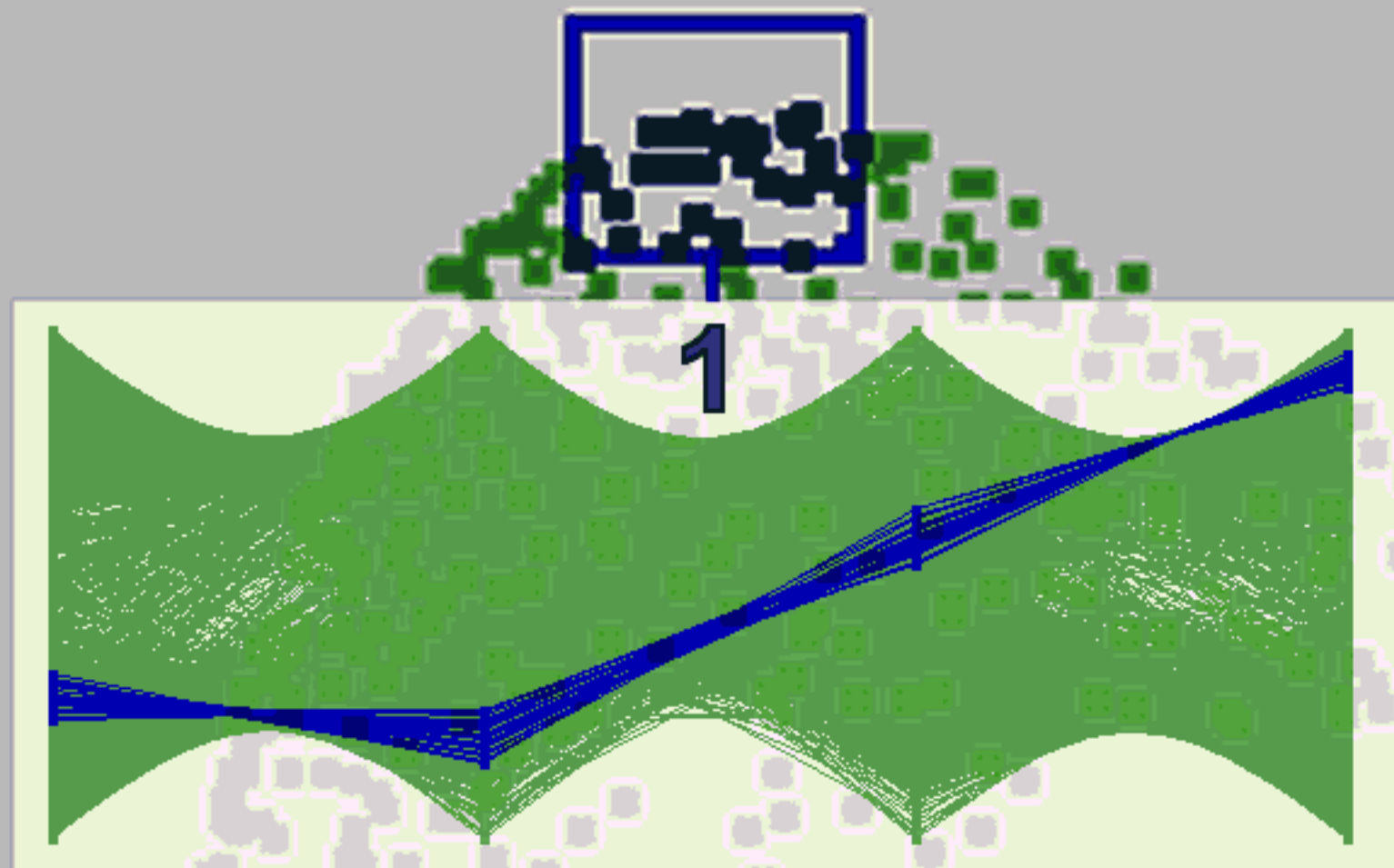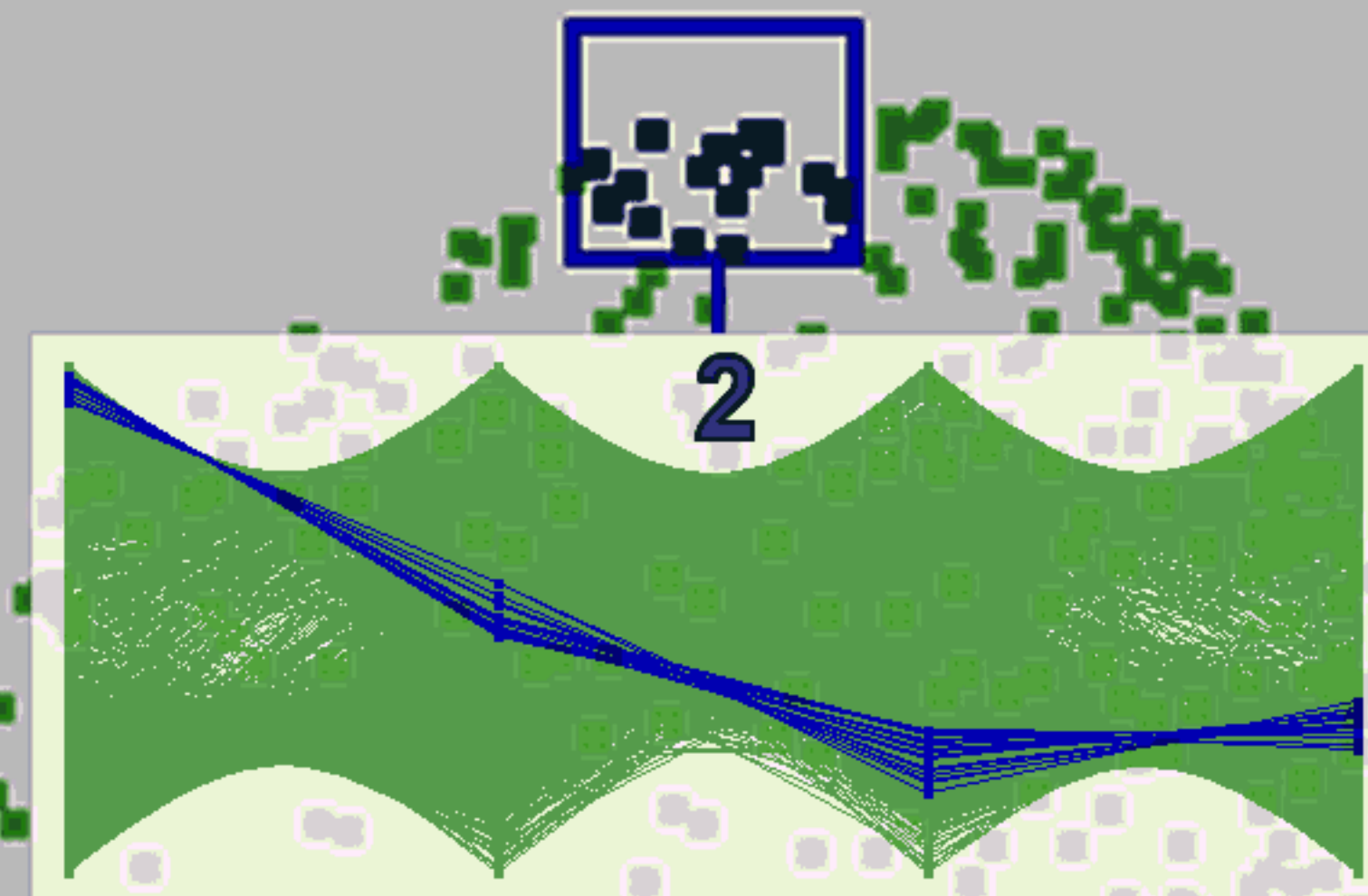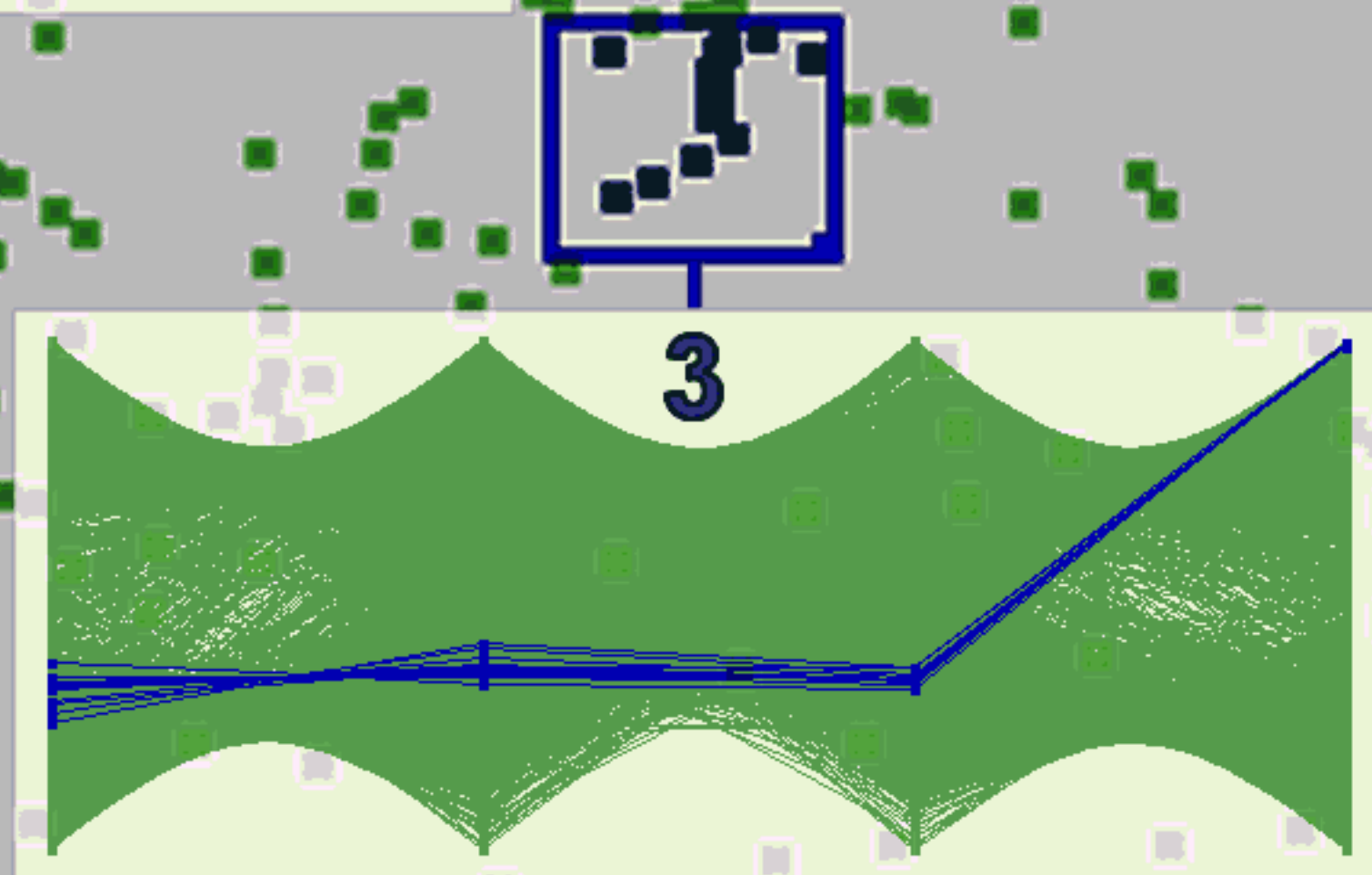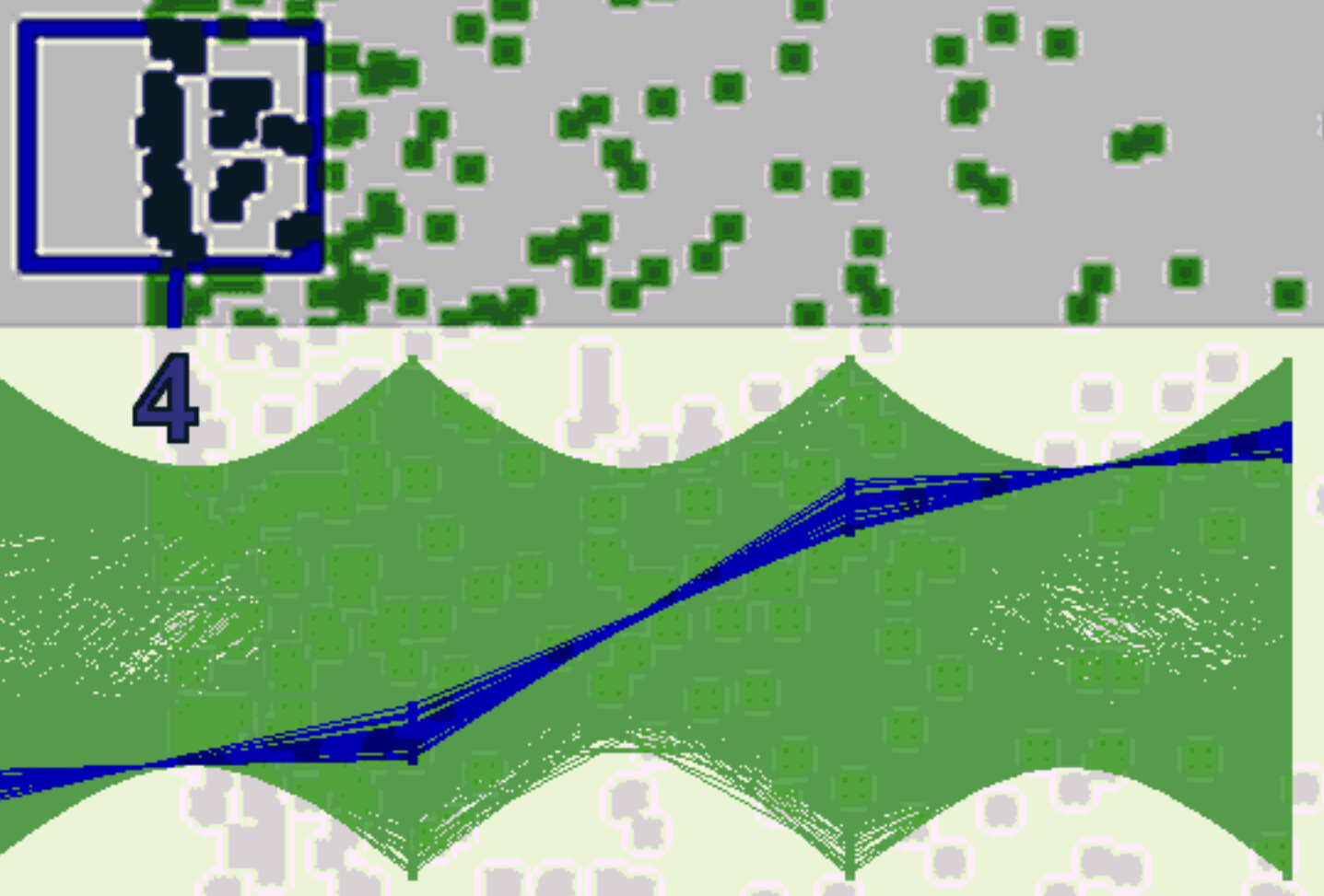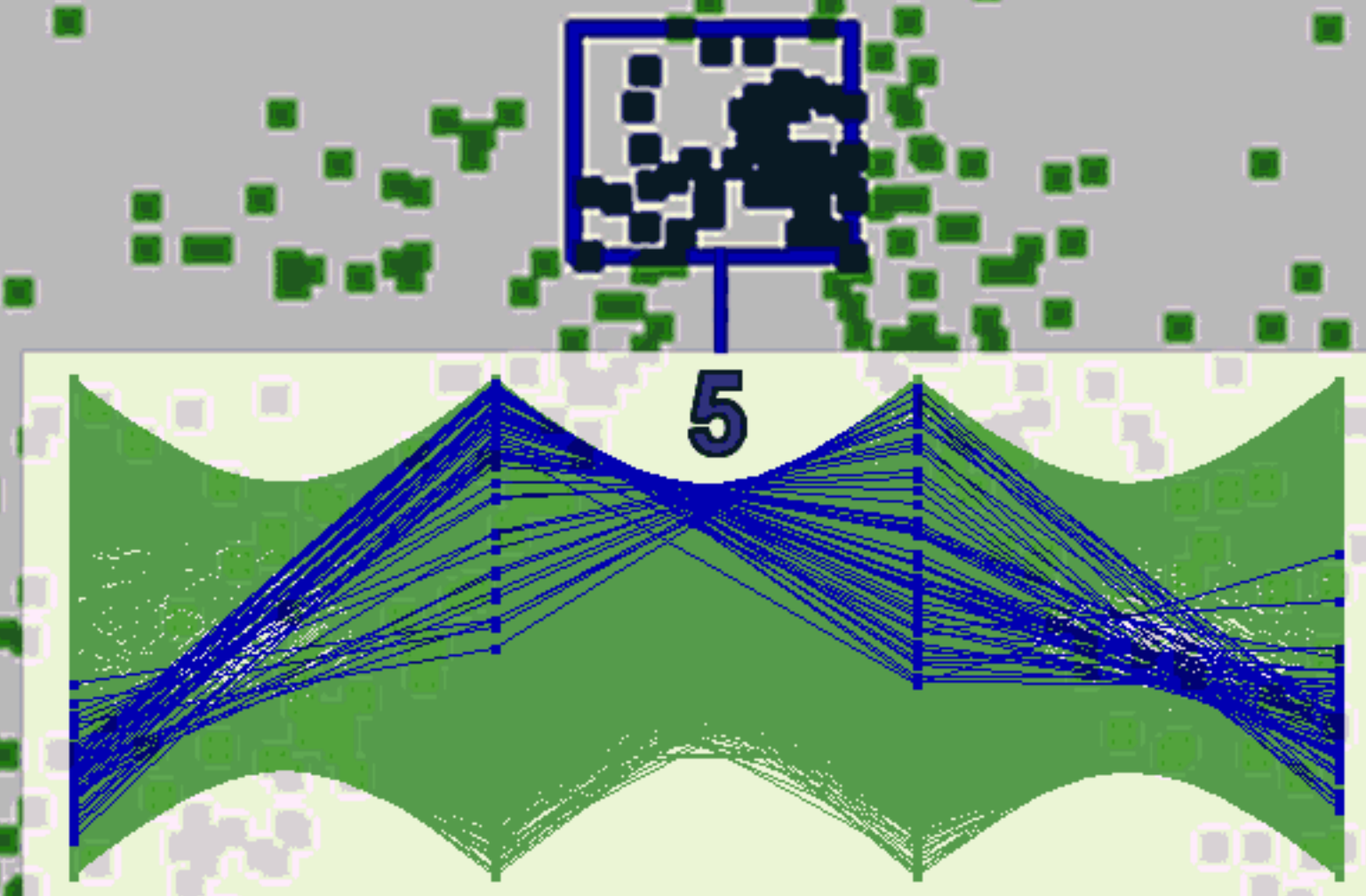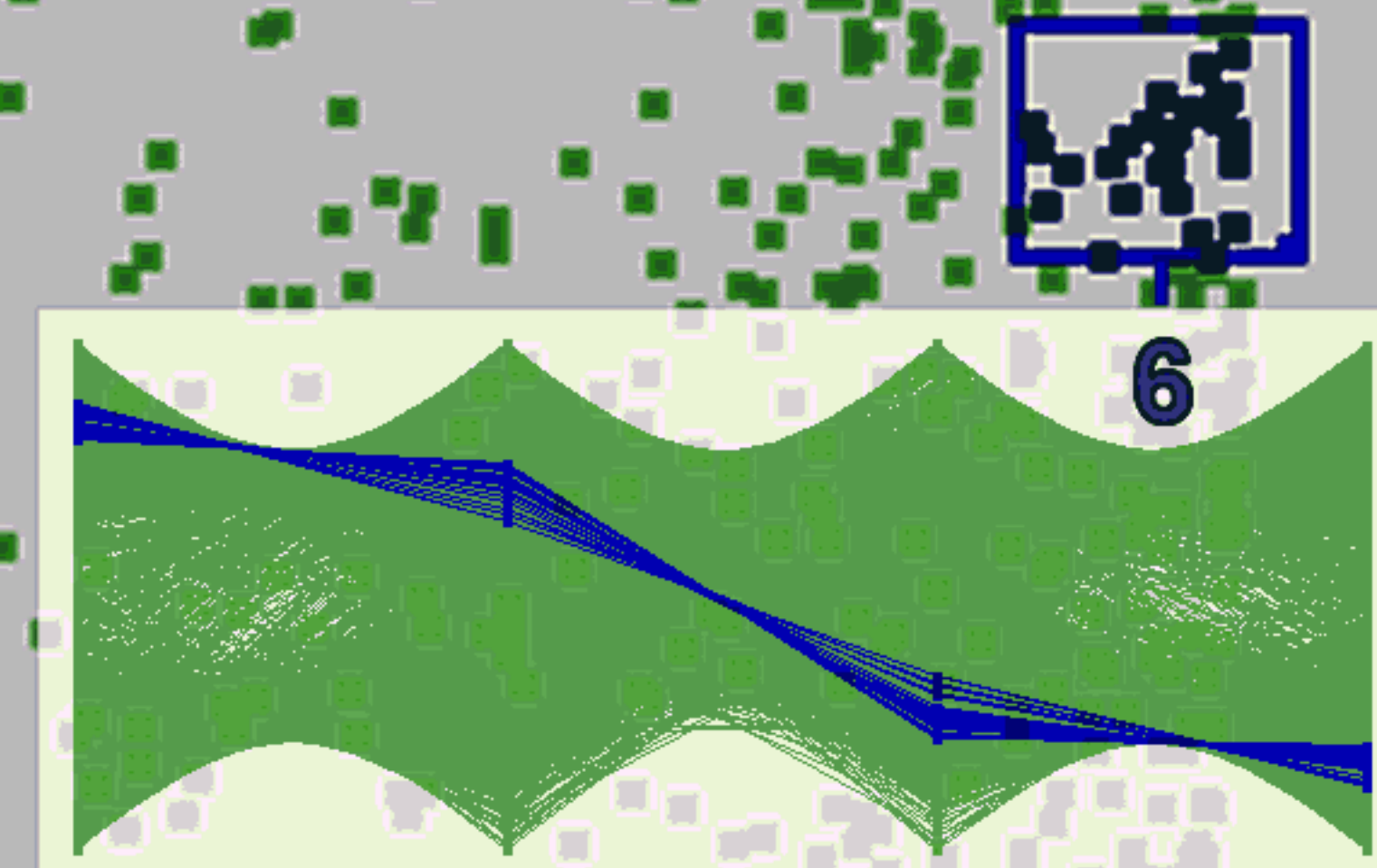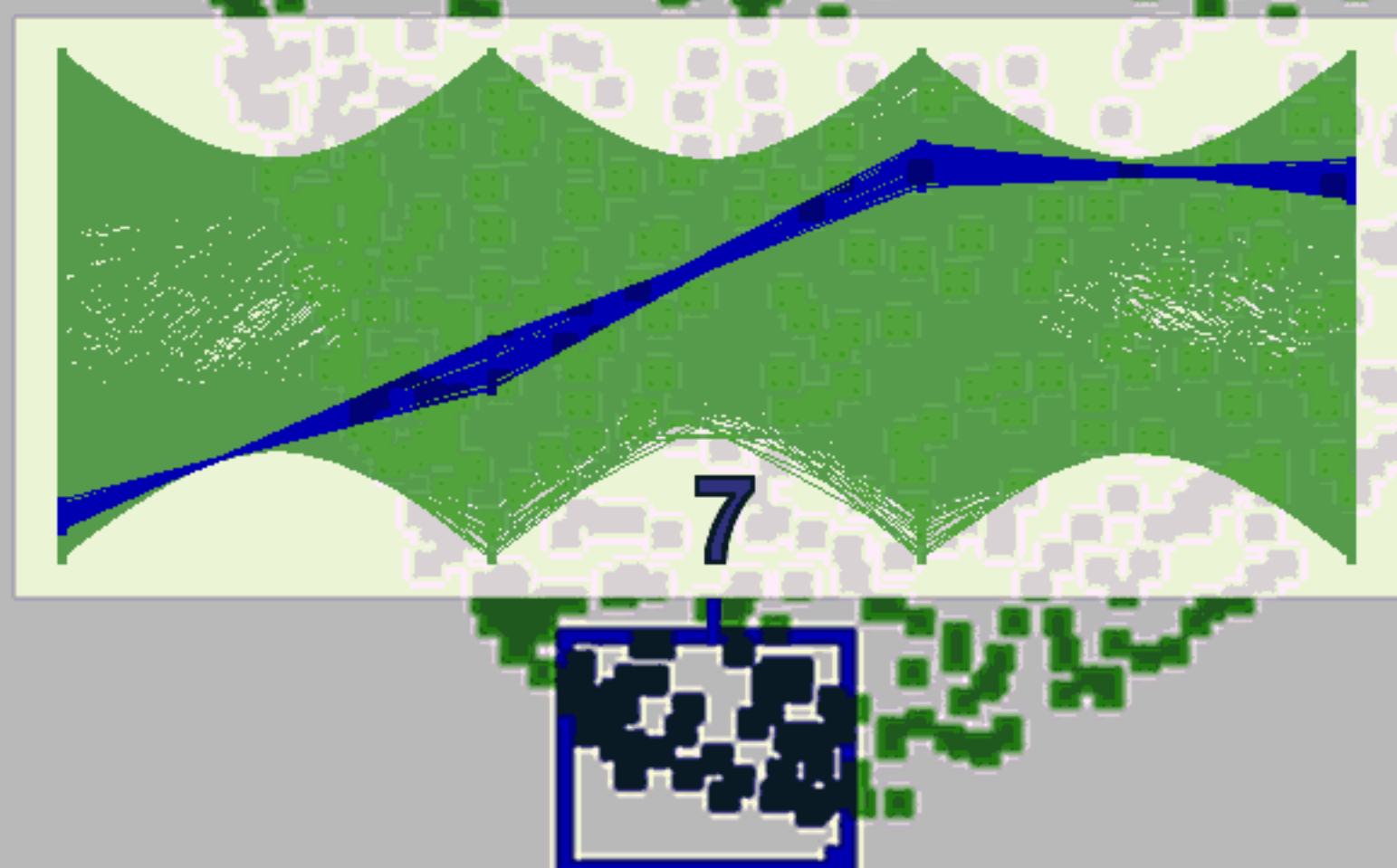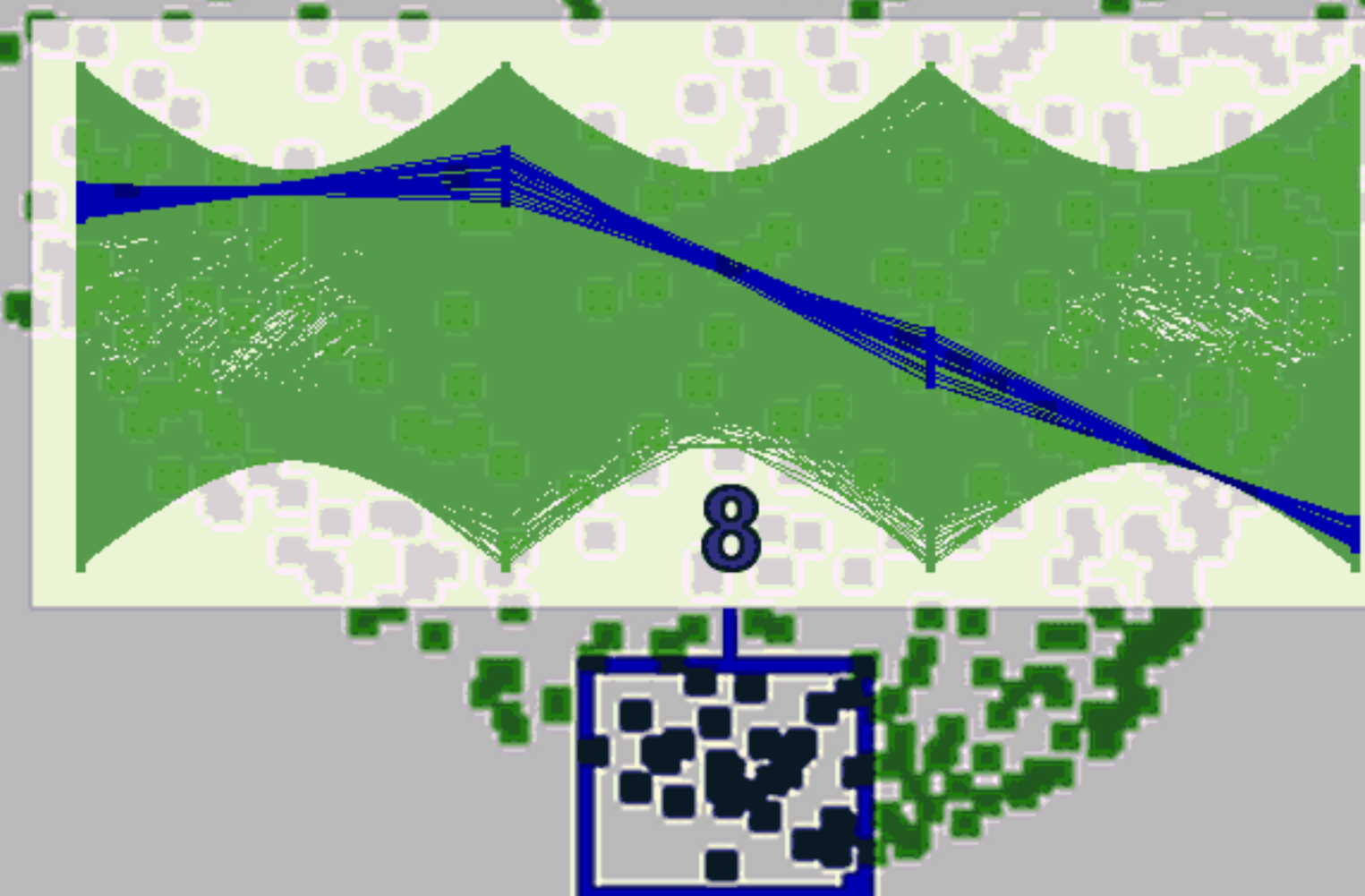

Supplement: Additional file 5 — Gene expression profiles obtained from Barley 1 Affymetrix gene chip connected to highlighted regions of the HiT-MDS-2 gene space plot [see Additional file 4]. High spatial specificity is observed in the exemplary clusters 1–8 covering interesting locations in the gene space. Patterns of up- and down-regulation fall into opposite poles. Cluster number 5 shows intermediate up-regulation with quite diverse characteristic, where Pearson correlation does not yield good discrimination between peaks in the second or third temporal stage. Cluster number 3 contains genes that become active just at the last stage (day 25 after flowering). [file 1471-2105-8-165-S5.pdf]

**Cluster: 2a**

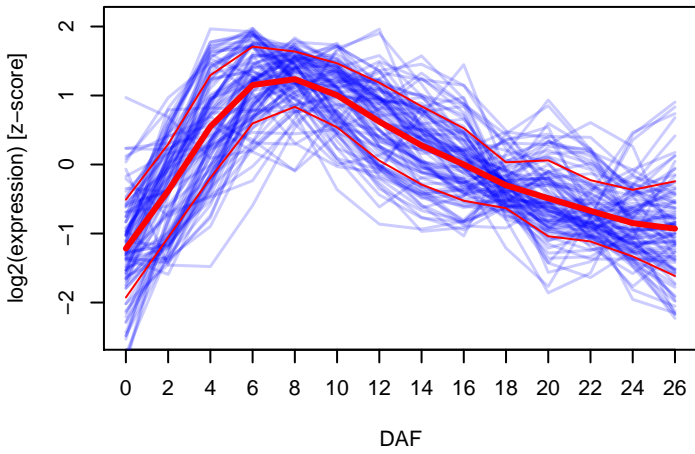

**Cluster: 2b**

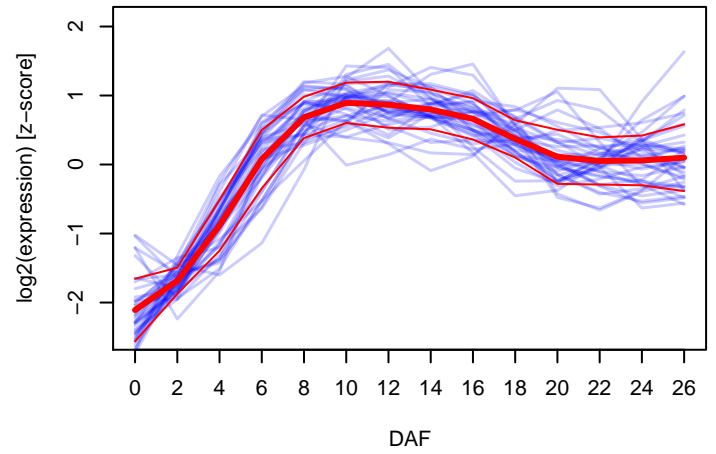

**Cluster: 3a**

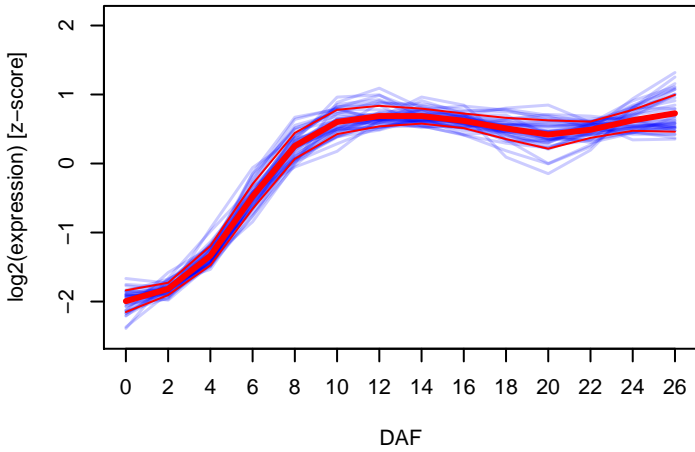

**Cluster: 3b**

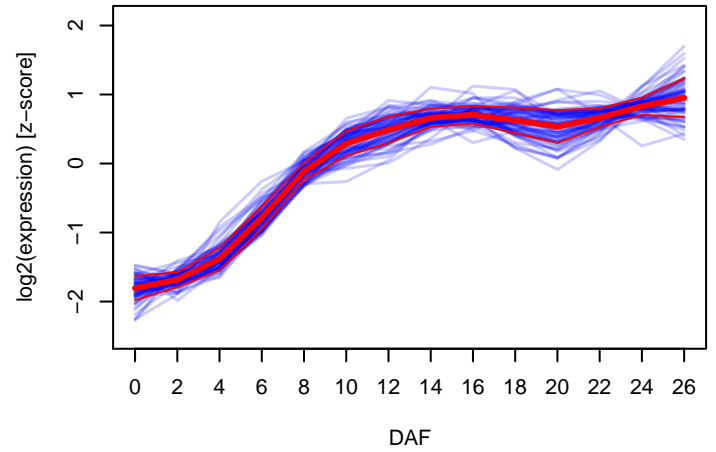

**Cluster: 4a**

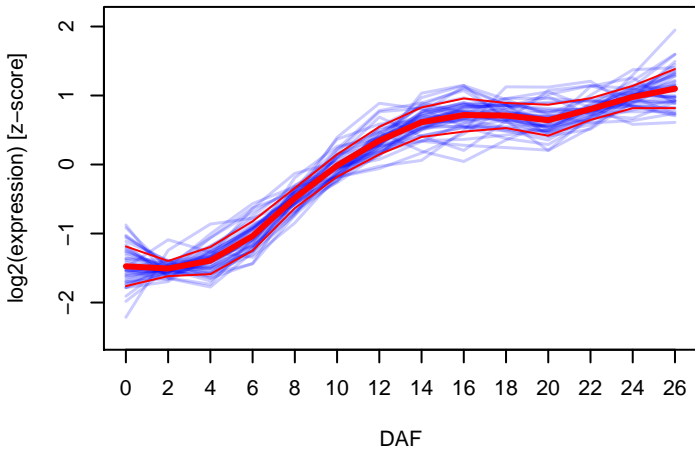

**Cluster: 4b**

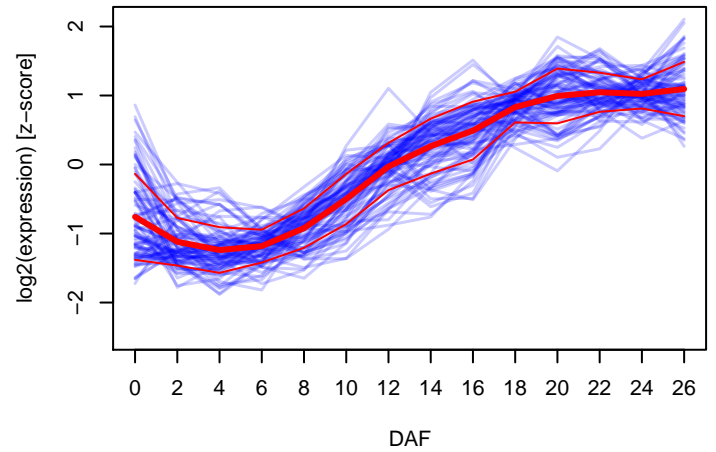

Supplement: Additional file 6 — Gene expression profiles corresponding to the six sub-clusters 2a, 2b, 3a, 3b, 4a and 4b referred in Figure 3 of the manuscript. The expression profiles reflect z-score normalized log2 values. In addition to individual gene expression curves displayed in blue, their mean and standard deviation are depicted by red lines. Highest variability is observed for intermediate regulation events in cluster 2a; yet, the overall quality of coexpression in the six clusters is represented well. [file 1471-2105-8-165-S6.pdf]
